# Estimating the clinical and economic burden of medically attended influenza in South Korea, stratified by age and comorbidity: A five-season hospital-based surveillance data, 2014/15–2018/19

**DOI:** 10.1371/journal.pone.0317643

**Published:** 2025-09-17

**Authors:** Min Joo Choi, Won Suk Choi, Joon Young Song, Hee Jin Cheong, Ji Yun Noh, Seong-Heon Wie, Jin Soo Lee, Jacob Lee, Hyo Youl Kim, Shin-Woo Kim, Kyong-Hwa Park, Woo Joo Kim

**Affiliations:** 1 Division of Infectious Diseases, Department of Internal Medicine, Korea University College of Medicine, Korea University Guro Hospital, Seoul, Republic of Korea; 2 Division of Infectious Diseases, Department of Internal Medicine, Korea University College of Medicine, Korea University Ansan Hospital, Ansan, Republic of Korea; 3 Vaccine Innovation Center, Korea University College of Medicine, Seoul, Republic of Korea; 4 Division of Infectious Diseases, Department of Internal Medicine, St. Vincent’s Hospital, Catholic University College of Medicine, Seoul, Republic of Korea; 5 Division of Infectious Diseases, Department of Internal Medicine, Inha University Hospital, Inha University College of Medicine, Incheon, Republic of Korea; 6 Division of Infectious Diseases, Department of Internal Medicine, Kangnam Sacred Heart Hospital, Hallym University College of Medicine, Seoul, Republic of Korea; 7 Division of Infectious Diseases, Department of Internal Medicine, Wonju Christian Hospital, Wonju College of Medicine, Yonsei University, Wonju, Republic of Korea; 8 Division of Infectious Diseases, Department of Internal Medicine, Kyungpook National University College of Medicine, Daegu, Republic of Korea; 9 Division of Infectious Diseases, Chonnam National University Medical School, Gwangju, Republic of Korea; Yonsei University Medical Center: Yonsei University Health System, KOREA, REPUBLIC OF

## Abstract

**Objectives:**

This study aims to estimate the clinical and economic burden of influenza in South Korea across five seasons (2014/15–2018/19), stratified by age and comorbidity, using data from the Hospital-based Influenza Morbidity and Mortality (HIMM) network.

**Methods:**

Data were gathered from eight university hospitals and included adults (≥ 20 years) with laboratory-confirmed influenza. The catchment population was estimated using hospital and national statistical data. Economic costs were evaluated from a societal perspective.

**Results:**

The incidence of medically attended influenza ranged from 113.1 to 220.7 per 100,000 adults, with hospitalization rate of 35.5 to 76.8, intensive care unit admission at 4.2 to 9.2, and deaths at 1.4 to 3.6. Annual socioeconomic costs ranged from $156 to $316 million, primarily driven by indirect costs associated with early mortality. The highest cost was recorded in 2017/18, following the largest outbreak, particularly among older adults. Per capita socioeconomic costs ranged from $2,747 to $4,072 across the seasons, with peak values observed in 2018/19 and 2015/16. Among at-risk groups, per capita costs were 1.5–2.3 times higher than those of the general adult population.

**Conclusions:**

Influenza imposes a significant clinical and economic burden on South Korea. Tailored strategies are essential to mitigate this burden, particularly among older adults and at-risk individuals not covered by the National Immunization Program.

## Introduction

Annual influenza epidemics contribute substantially to global morbidity and mortality, particularly among older adults and those with underlying chronic conditions [1,2]. From 1999 to 2015, global estimates indicate that 290,000–650,000 influenza-related deaths occur annually, corresponding to 4.0–8.8 deaths per 100,000 individuals overall and 51.3–99.4 per 100,000 individuals aged 75 years or older [1]. The risk of severe outcomes is exacerbated by coinfection with other respiratory pathogens, underscoring the need for timely influenza vaccination and other preventive measures [3].

Although the global burden of influenza is well recognized, its regional impact varies substantially, requiring tailored public health strategies [4]. In Asia, however, data on the actual influenza burden are limited. Existing studies often cover only short periods [5–8], rely on small sample sizes (<200 participants) [7,9], or are based data collected prior to 2009. Even globally, few studies have comprehensively assessed both the clinical and economic burdens of influenza over multiple seasons, often relying on claim data that may not accurately reflect diagnoses or outcomes [10–15].

The recent COVID-19 pandemic has underscored the need for prioritizing respiratory virus surveillance and vaccination strategies. With the continued circulation of COVID-19, the seasonal impact of influenza, and the recent introduction of respiratory syncytial virus vaccines, more comprehensive data is needed to guide evidence-based prioritization of vaccine development, implementation, and resource allocation across competing respiratory pathogens [3,16,17]. Reliable burden estimates, particularly among high-risk groups, serve as a critical foundation for these evaluations and policy decisions.

This study sought to estimate the nationwide burden of influenza in South Korea—a temperate Asian country—across five consecutive seasons (2014/15–2018/19) using individual-level data from the Hospital-based Influenza Morbidity and Mortality (HIMM) surveillance network. The analysis encompassed clinical outcomes and economic costs, stratified by age and comorbidity status, to provide actionable evidence for influenza control and healthcare planning.

## Materials and methods

### Study sites and participants

The HIMM surveillance system, established in 2011, monitors influenza cases across emergency rooms and inpatients [18]. The system expanded to outpatient surveillance in 2017. Briefly, for Emergency Room or outpatient-based surveillance, informed consent was obtained from patients with influenza-like illness, defined as a sudden onset of fever (≥ 37.8°C) accompanied by at least one respiratory symptom (cough, sore throat, or rhinorrhea), followed by nasal/throat swab collection. Inpatient-based surveillance involved the clinical evaluation and monitoring of influenza-related hospitalization, intensive care unit (ICU) care, and death from any recent (< 4 weeks) laboratory-confirmed influenza (LCI).

During the study period, eight university hospitals were selected across South Korea, considering geographic distribution and accessibility: the Seoul metropolitan area (Seoul, Ansan, and Incheon), Gangwon region (Wonju), Jeolla region (Gwangju), Chungcheong region (Daejeon), and Gyeongsang region (Daegu) (S1 Fig in S1 File). Although more study sites were located within the capital area, this reflects the actual population distribution in Korea, with over 50% of the population residing in the Seoul metropolitan region [19].

Individuals aged ≥ 20 years who visited the participating hospitals with LCI during five seasons (2014/15–2018/19) were included. The duration of each season was defined as spanning from September of one year to May of the subsequent year. LCI was diagnosed when influenza virus infection was confirmed in respiratory specimens using at least one of the following methods: rapid antigen testing (RAT), polymerase chain reaction (PCR), or virus culture. The choice of diagnostic method was made by the attending physician, based on clinical judgment and institutional protocols.

The analysis also included hospital-acquired influenza, defined as cases with symptom onset occurring 48 h or more after admission for a different condition. To avoid overestimating healthcare resource use in these cases, the duration of hospitalization and associated costs were conservatively estimated using a standard five-day course of antiviral treatment.

### Collection of clinical information

Trained personnel reviewed medical records to gather patient information, including age, sex, comorbidities, vaccination history, diagnostic methods, influenza type/subtype, symptom onset, emergency room and outpatient visits, complications, hospitalization, ICU admission, antiviral use, and influenza-related deaths. Only deaths directly attributable to influenza were considered, as determined by infectious disease specialists at each hospital through a thorough review of the deceased patients’ medical records.

### Estimation of cost

The economic burden was evaluated from a societal perspective. Direct costs comprised medical expenses (hospitalization, outpatient visits, and prescribed medications) and non-medical expenses (transportation and nursing care). Indirect costs included productivity loss due to illness or early death. Detailed information on cost components and calculation formulas can be found in the Supplementary Methods (S1, S2 Tables in S1 File). Costs were converted from Korean won to US dollars (1 USD = 1,100 KRW), based on the average exchange rate during the study period [19]. Indirect costs due to productivity loss were calculated using two methods: one including all adults and another excluding individuals aged ≥ 65 years. The primary results considered productivity loss for all adults, including individuals aged ≥ 65 years, to reflect the substantial labor force participation in this age group (approximately 30%) and to capture the full economic impact in an aging society [19].

### Data analysis

Categorical variables are presented as numbers and percentages, while continuous variables are reported as medians with interquartile ranges to address potential skewness. The incidences of LCI and related events (admission, ICU admission, and death) were calculated using the catchment population based on annual patient visits [19,20]. Additional details, including stratification by age and comorbidity, are provided in the supplementary materials (S3 Table in S1 File). Outcomes are presented by age groups (20–49, 50–64, 65–74, and ≥ 75 years) and by comorbidity type for at-risk individuals.

The study was conducted in accordance with the Declaration of Helsinki and was approved by the Institutional Review Boards of Korea University Guro Hospital (2019GR0475), Korea University Ansan Hospital (2020AS0045), Catholic University St. Vincent’s Hospital (VC20RIDI0056), Inha University Hospital (2020-01-013), Hallym University Kangnam Sacred Hospital (2020-01-004), Wonju Christian Hospital (CR320008), Kyungpook National University Hospital (KNUH 2020-03-042), and Chonnam National University Hospital (CNUH-2020–064). Written informed consent was waived due to the retrospective nature of the study. Medical records were accessed for research purposes at different times for each study site: Korea University Guro Hospital from 1 January 2020–31 December 2020; Korea University Ansan Hospital, Hallym University Kangnam Sacred Hospital, and Inha University Hospital from 15 February 2020–31 December 2020; and Wonju Christian Hospital, Chonnam National University Hospital, Catholic University St. Vincent’s Hospital, and Kyungpook National University Hospital from 15 April 2020–31 December 2020. Identifiable information was accessed during data collection to ensure accurate data extraction, in accordance with the protocol approved by the Institutional Review Boards. All data were anonymized before being transferred outside each hospital, ensuring participant confidentiality.

## Results

### Characteristics of medically attended laboratory-confirmed influenza in eight hospitals

Across five seasons, a total of 16,514 LCI cases were recorded, with 2,503–4,867 cases of per season, peaking in 2017/18, followed by 2018/19 (Table 1). The predominant influenza type varied by season, with influenza A responsible for 54–90% of cases. Influenza B contributed to approximately 30% of cases in the 2014/15 and 2017/18 seasons. Most sought medical attention within three days of symptom onset, with over 90% diagnosed via rapid antigen tests. Approximately 40% of cases were male. The median age across all seasons was 53 years (seasonal ranged, 45–58 years), while the at-risk group was older, with a median age of 68 years (seasonal range, 63–70 years) (S4 Table in S1 File). At-risk individuals comprised 53% of all cases (seasonal range, 38–54%), with hypertension (25%; 19–29%) and diabetes (14%; 11–17%) being the most common comorbidities. Other common conditions included chronic cardiovascular diseases, chronic pulmonary diseases, and immunodeficiency (each 9–10%). Overall, 30% of patients were vaccinated at least 14 days before symptom onset (seasonal range 29–46%), with vaccination rates increasing with age: 19% (17–23%) in those aged 20–49, 26% (23–33%) in 50–64, 69% (64–68%) in 65–74, and 75% (68–80%) in those ≥ 75 years (S5 Table in S1 File). Similar rates were observed in those with comorbidities (S4 Table in S1 File).

**Table 1:** Baseline characteristics of laboratory-confirmed influenza cases at eight participating hospitals.

	2014/15 (*n* = 2,957)	2015/16 (*n* = 2,652)	2016/17 (*n* = 2,503)	2017/18 (*n* = 4,867)	2018/19 (*n* = 3,535)
Symptom onset to seeking medical attention, median (Q1-Q3), days^a^	2 (1–3)	1 (1–3)	1 (0–3)	1 (0–3)	1 (0–2)
Diagnosis method, no. (%)					
RAT	2,708 (91.6)	2429 (91.6)	2,315 (92.5)	4,422 (90.9)	3,261 (92.2)
PCR	507 (17.1)	432 (16.3)	433 (17.3)	803 (16.5)	450 (12.7)
Culture	19 (0.6)	4 (0.2)	0	0	1 (<0.1)
Influenza subtype, no. (%)					
Influenza A/H3N2	85 (2.9)	1 (< 0.1)	74 (3.0)	361 (7.4)	50 (1.4)
Influenza A/H1N1	66 (2.2)	52 (2.0)	2 (0.1)	19 (0.4)	284 (8.0)
Influenza A/unknown	1978 (66.9)	2158 (81.4)	2,263 (90.4)	2,642 (54.3)	2686 (76.0)
Influenza B	835 (28.2)	458 (17.3)	164 (6.6)	1,824 (37.5)	513 (14.5)
Both A & B	7 (0.2)	17 (0.6)	4 (0.2)	21 (0.4)	2 (0.1)
Age, median (Q1-Q3), years	54 (37–72)	46 (34–63)	54 (34–71)	58 (39–74)	45 (31–64)
20–49 years, no. (%)	1,262 (42.7)	1,447 (54.6)	1,117 (44.6)	1,755 (36.1)	1,959 (55.4)
50–64 years, no. (%)	670 (22.7)	623 (23.5)	561 (22.4)	1,241 (25.5)	702 (19.9)
65–74 years, no. (%)	436 (14.7)	278 (10.5)	347 (13.9)	711 (14.6)	383 (10.8)
≥ 75 years, no. (%)	589 (19.9)	304 (11.5)	478 (19.1)	1,160 (23.8)	491 (13.9)
Male, no. (%)	1,162 (39.3)	1,037 (39.1)	1,028 (41.1)	2,102 (43.2)	1,521 (43)
Influenza vaccination, no. (%)^b^	888 (41.4)	646 (29.4)	808 (41.2)	1,807 (46.3)	825 (34.5)
Comorbidities ≥ 1, no. (%)	1,439 (48.7)	1,032 (38.9)	1,248 (49.9)	2642 (54.3)	1,348 (38.1)
Diabetes	432 (14.6)	289 (10.9)	416 (16.6)	806 (16.6)	440 (12.4)
Hypertension	779 (26.3)	511 (19.3)	672 (26.8)	1,428 (29.3)	671 (19.0)
Chronic cardiovascular disease (except hypertension)	294 (9.9)	186 (7.0)	279 (11.1)	639 (13.1)	303 (8.6)
Chronic pulmonary disease	312 (10.6)	133 (5.0)	239 (9.5)	515 (10.6)	224 (6.3)
Chronic renal disease	151 (5.1)	108 (4.1)	137 (5.5)	313 (6.4)	157 (4.4)
Chronic liver disease	88 (3.0)	76 (2.9)	69 (2.8)	181 (3.7)	99 (2.8)
Cerebrovascular disease	206 (7.0)	132 (5.0)	215 (8.6)	491 (10.1)	243 (6.9)
Neuromuscular disease	40 (1.4)	40 (1.5)	46 (1.8)	107 (2.2)	55 (1.6)
Autoimmune disease	43 (1.5)	60 (2.3)	51 (2.0)	120 (2.5)	47 (1.3)
Immunocompromised^c^	261 (8.8)	229 (8.6)	287 (11.5)	620 (12.7)	351 (9.9)
Pregnancy, no. (%)	70 (2.4)	62 (2.3)	69 (2.8)	87 (1.8)	98 (2.8)
Coinfection with other RV, no. (%)^d^	27 (2.9)	35 (7.3)	33 (5.5)	87 (10.5)	49/661 (7.4)
RSV	11 (1.2)	8 (1.7)	7 (1.2)	20 (2.4)	9 (1.4)
HRV	5 (0.5)	9 (1.9)	12 (2.0)	23 (2.8)	17 (2.6)
AdV	5 (0.5)	5 (1.0)	1 (0.2)	2 (0.2)	2 (0.3)
Coronavirus 229E	0	6 (1.3)	0	23 (2.8)	1 (0.1)
Coronavirus OC43/HKU1	1 (0.1)	3 (0.6)	3 (0.5)	16 (1.9)	6 (0.9)
Coronavirus NL63	1 (0.1)	1 (0.2)	4 (0.7)	2 (0.2)	10 (1.5)
Bocavirus	1 (0.1)	0	0	2 (0.2)	4 (0.6)
MPV	3 (0.3)	2 (0.4)	2 (0.3)	0	2 (0.3)
Parainfluenza virus	0	2 (0.4)	2 (0.3)	3 (0.4)	2 (0.3)
Enterovirus	0	0	2 (0.3)	3 (0.4)	0

IQR, interquartile range; RAT, rapid antigen test; PCR, polymerase chain reaction; no, number; SD, standard deviation; RV, respiratory virus; RSV, respiratory syncytial virus; HRV, human rhinovirus, AdV, adenovirus; MPV, metapneumovirus.

^a^Duration from symptom onset to initial hospital visit, excluding cases with in-hospital onset.

^b^Influenza vaccine recipients during a specific season, including only those who received the vaccine 14 days or more before symptom onset. When calculating the ratio, individuals with unknown vaccination statuses were excluded.

^c^Solid organ cancer, hematological malignancies, bone marrow or organ transplant recipients, HIV infection, and individuals undergoing immunosuppressive therapy.

^d^Ratio calculation excluded individuals not tested for concurrent infections with other respiratory viruses.

Baseline characteristics of fatal cases are presented in S6 Table in S1 File. In most seasons, influenza A accounted for ≥ 90% of fatalities, with A/H3N2 and A/H1N1 predominant in 2017/18 and 2018/19, respectively. During the 2014/15 and 2017/18 seasons, individuals aged ≥ 65 accounted for over 80% of deaths, with influenza B contributing significantly. Comorbidities were present in 78–98% of those who died. S7 Table in S1 File summarizes the factors independently associated with mortality: delayed presentation (≥ 3 days after symptom onset; adjusted odds ratio [aOR]: 1.46, 95% confidence interval [CI]: 1.06–2.00), older age (≥ 65 years; aOR: 7.97, 95% CI: 4.76–13.35), presence of comorbidity (aOR: 4.93, 95% CI: 2.65–9.19), and influenza vaccination (aOR: 0.57, 95% CI: 0.41–0.80).

### Estimated incidence of influenza and related outcomes stratified by age and comorbidities

**Fig 1:**
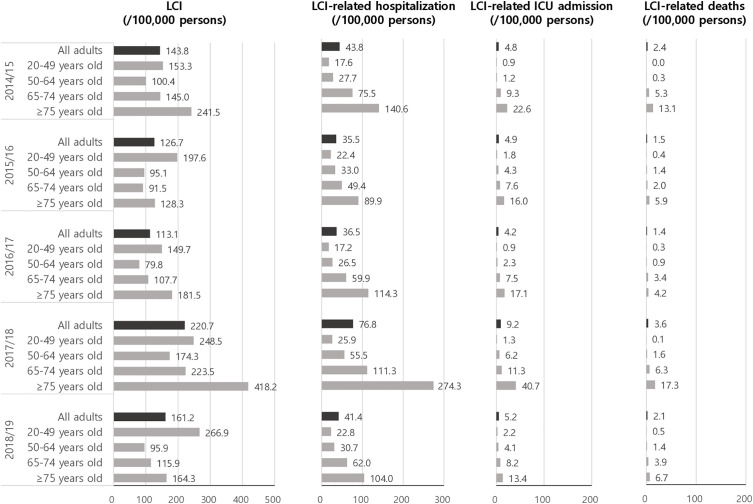
Estimated incidence per 100,000 persons of laboratory-confirmed influenza and related morbidity and mortality rates.

The incidence rate of LCI ranged from 113.1 to 220.7 per 100,000 individuals across the 2014/15–2018/19 seasons. The highest incidence was observed in 2017/18, followed by 2018/19. Across most seasons, the 20–49 age group had the highest incidence; however, the highest rates occurred in individuals aged ≥ 75 during 2014/15 and 2017/18 (Fig 1). Hospitalization, ICU admission, and mortality rates per 100,000 individuals increased with age. Influenza-related deaths varied between 1.4 and 3.6 per 100,000 individuals overall, and between 4.2 and 17.3 per 100,000 in those aged ≥ 75. In at-risk populations, age-specific influenza incidence was comparable to the general adult population, while hospitalization, ICU admission, and death rates were generally higher (Fig S2 in S1 File). Those with chronic kidney disease or immunocompromised conditions exhibited higher frequencies of influenza and related complications compared to those with other comorbidities.

### Estimated clinical burden and severity of medically attended laboratory-confirmed influenza

**Fig 2:**
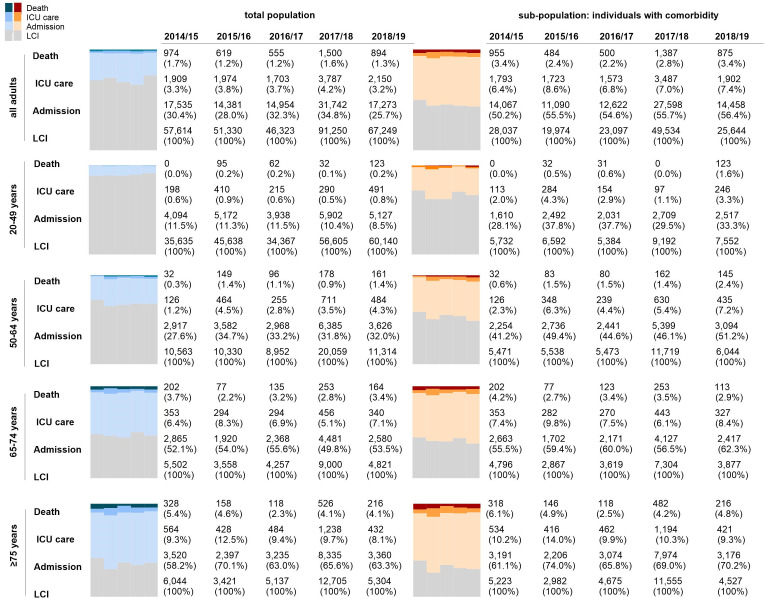
Severity of laboratory-confirmed influenza during five influenza seasons (2014/15–2018/19).

The clinical course of LCI cases is detailed in S8 and S9 Tables in S1 File, with nationwide estimates in Fig 2. Hospitalization, ICU admission, and mortality rates for LCI cases were 26–35%, 3–4%, and 1.2–1.7%, respectively. These outcomes increased significantly with age and were consistently higher among individuals with comorbidities. Mortality rates were notably higher during the 2014/15 and 2017/18 seasons, particularly affecting older adults (≥ 65 years). In 2018/19, severity was notably higher among at-risk individuals aged 20–64, with ICU admission and mortality rates up to 6.7 and 2.4 times higher, respectively, than in other seasons. The median hospital stay remained consistent at 5–6 days, increasing with age, while ICU stay ranged from 5 to 7 days. Antiviral medications were prescribed in over 90% of cases across all age groups and seasons.

In fatal cases, complications were more frequent (> 85%) and antiviral use was generally lower than in the overall study population (S6 Table in S1 File).

### Estimation of socioeconomic cost

**Fig 3:**
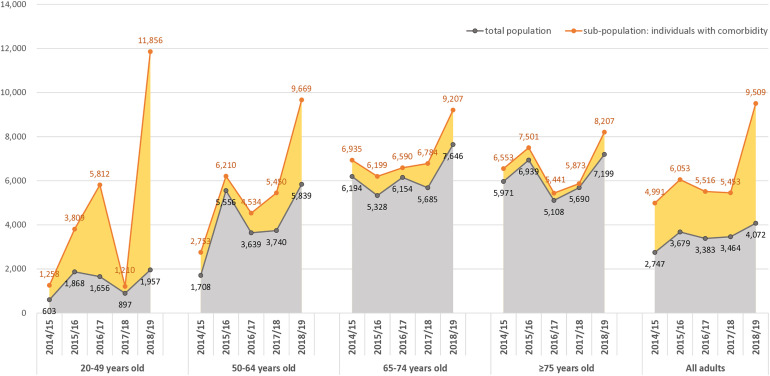
Analysis of per capita socioeconomic costs based on age and comorbidities.

The average per capita direct medical cost, which includes both outpatient and inpatient components, ranged from $1,165 to $1,670, with the highest values in the 2018/19 and 2015/16 seasons and the lowest in 2014/15 (S3 Fig in S1 File, Table 2). Outpatient costs per capita varied between $286 and $339, while inpatient costs spanned from $3,174 to $5,520. The total socioeconomic costs, encompassing transportation ($7.1–12.1 million), nursing care ($5.8–12.2 million), indirect costs resulting from hospital visits ($5.4–10.7 million), and early death ($70.1–142.3 million), ranged from $156.7 to 316.1 million. The highest total costs were recorded during the 2017/18 season, followed by 2018/19 and 2015/16. Indirect costs attributed to early death comprised the largest portion of the total burden (44–51%), followed by direct medical costs (41–44%). Conversely, per capita socioeconomic costs were highest during 2018/19 and 2015/16, primarily driven by high direct medical costs and significant indirect costs from early death (Fig 3, S10 Table in S1 File).

**Table 2:** Annual average cost per capita.

	Estimated cost (USD)^a^	2014/15	2015/16	2016/17	2017/18	2018/19
**All adults**	Direct medical cost (/person)	1,165 (42.4%)	1,523 (41.4%)	1,382 (41.1%)	1,520 (43.9%)	1,670 (41.0%)
	Direct non-medical costs (/person)					
	Transportation	139 (5.1%)	143 (3.9%)	157 (4.7%)	133 (3.8%)	106 (2.6%)
	Nursing	113 (4.1%)	115 (3.1%)	136 (4.1%)	133 (3.9%)	115 (2.8%)
	Indirect costs (/person)^b^					
	Due to visiting clinic or admission	113 (4.1%)	132 (3.6%)	117 (3.5%)	117 (3.4%)	117 (2.9%)
	Due to early death	1,217 (44.3%)	1,765 (48.0%)	1,570 (46.7%)	1,560 (45.0%)	2,064 (50.7%)
	Total costs (/person)	2,747	3,679	3,383	3,464	4,072
	Total costs (nation-level)^c^	158,259,104	188,818,353	156,719,915	316,056,600	273,831,727
**Sub-population:** **Individuals with comorbidity**	Direct medical cost (/person)	1,940 (38.9%)	2,826 (46.7%)	2,306 (41.8%)	2,333 (42.8%)	3,570 (37.5%)
	Direct non-medical costs					
	Transportation (/person)	220 (4.4%)	264 (4.4%)	259 (4.7%)	203 (3.7%)	208 (2.2%)
	Nursing (/person)	200 (4.0%)	240 (4.0%)	247 (4.5%)	221 (4.1%)	262 (2.8%)
	Indirect cost (/person)^b^					
	Due to visiting clinic or admission	156 (3.1%)	220 (3.6%)	177 (3.2%)	166 (3.0%)	211 (2.2%)
	Due to early death	2,476 (49.6%)	2,502 (41.3%)	2,526 (45.8%)	2,529 (46.4%)	5,258 (55.3%)
	Total costs (/person)	4,991	6,053	5,516	5,453	9,509
	Total costs (nation-level)^c^	139,926,020	120,899,473	127,393,716	270,109,877	243,837,667

^a^USD $1 = KRW ₩1,100.

^b^All adults (including older adults) were presumed to have experienced a loss in productivity.

^c^The social perspective costs obtained from the eight hospitals were extrapolated to the nationwide level by multiplying the cost by the ratio of the nationwide annual patient visits to that of the eight participating hospitals.

Age group analysis yielded similar results (Fig 3, S10 Table in S1 File); however, for individuals aged 20–49, the direct medical and indirect costs from early death were significantly higher in the 2018/19 season, leading to the highest per capita and overall socioeconomic costs. At-risk individuals had per capita socioeconomic costs 1.5–2.3 times higher than the general population (Fig 3, S11 Table in S1 File). Despite slight variations across chronic conditions, per capita costs remained high during the 2018/19 season (S12 Table, S4 Fig in S1 File).

Excluding productivity loss for individuals aged ≥ 65 years, the annual socioeconomic cost ranged from $88 to $199 million, with direct medical costs contributing 56–76%, and indirect costs from early death 15–32%. In the 2014/15 season, transportation costs were the second-largest contributor at 9% (S13 Table, S5 Fig in S1 File).

## Discussion

The burden of influenza in South Korea is considerable, with seasonal and subgroup-specific variations influenced by circulating strains, age, comorbidities, and vaccination rates. During this study, the incidence of medically attended influenza ranged from 113.1 to 220.7 per 100,000, with hospitalization rates between 35.5 and 76.8, ICU admissions between 4.2 and 9.2, and mortality between 1.4 and 3.6 per 100,000 adults. Annually, 2,530–4,867 patients were recorded in the HIMM surveillance, extrapolated to 46,323–91,250 nationwide. Mortality was highest during the 2014/15 and 2017/18 seasons, particularly among older adults, while ICU admissions were more frequent in younger adults with comorbidities during the 2015/16 and 2018/19 seasons. Total socioeconomic costs ranged from $156 to $316 million, peaking in 2017/18. Per capita costs ranged from $2,747 to $4,072, peaking in 2018/19, followed by 2015/16. Costs for at-risk individuals were 1.5–2.3 times higher than those for the general population.

The results of this study align closely with previous Korean estimates based on the same data source. A previous analysis of the 2013/14 season using the HIMM network indicated an incidence rate of 242.8 per 100,000 adults and a mortality rate of 3.1 per 100,000 adults [20], falling within the seasonal range observed in our study. However, our incidence estimates were slightly lower than those derived from nationwide claims data covering 2010–2020, particularly among younger adults, with seasonal differences ranging from twofold to over tenfold [21]. These discrepancies are likely due to differences in surveillance scope and case definitions: our hospital-based system captures laboratory-confirmed, medically-attended adult cases, whereas claims data encompass clinically suspected episodes across all age groups. The use of calendar-year data in the claims study and differing age categorization also limit direct comparability. Although more conservative, our approach avoids the potential overestimation of ICD code-based data. Despite not all patients undergoing testing, HIMM hospitals actively conduct diagnostic testing for influenza-like illness, thus reducing the likelihood of missed cases. Importantly, even with lower incidence estimates, age-specific mortality rates in our study are consistent with national claims data, indicating that severe cases are effectively captured through hospital-based surveillance. This underscores the utility of HIMM data for monitoring the clinical and economic burden of influenza in Korea.

The estimated average socioeconomic cost of influenza per Korean adult was equivalent to 10% of the GDP per capita in South Korea, highlighting its considerable national burden [19]. While absolute costs vary by country, international estimates consistently indicate a substantial burden relative to each country’s economic context: annual socioeconomic costs of $11.2 billion in the US, €141 million in France, and $169 million in Bangladesh have been reported [7,10,22]. Per-case hospitalization costs were $7,800–$11,900 in the US, $11,444 in the Canada, and $5,400–$9,800 in Netherlands and Japan–figures comparable to those in our study [6,15,22,23]. China and Bangladesh reported lower estimates ($1,100–$1,800 and $82, respectively) [7,8]. Despite demographic, economic, and health system differences, these figures highlight the significant burden of influenza across countries and the need for sustained surveillance and proactive prevention strategies tailored to national priorities.

Importantly, older adults in our study experienced the highest influenza-related mortality rates, aligning with global evidence of exponentially rising death rates with advancing age [1]. Notably, during the 2014/15 and 2017/18 seasons, the incidence among adults aged ≥ 75 years exceeded that of younger adults. This coincided with markedly elevated hospitalization and mortality rates in this demographic. During these seasons, South Korea experienced co-circulating A/H3N2 and B strains (S14 Table in S1 File), with significant outbreaks among older adults reported globally, partly attributed to vaccine strain mismatches [24–26]. In particular, the 2017/18 season witnessed the largest outbreak within our study, resulting in the highest socioeconomic costs. Although vaccination coverage among Korean seniors was relatively high during this season, the predominant B/Yamagata lineage was not included in the available trivalent vaccines, likely contributing to reduced protection and increased outbreak severity [27]. Beyond vaccine mismatch, factors such as age-related immunosenescence and a higher prevalence of chronic diseases may have contributed to increased vulnerability in this group [28]. Hence, even in well-vaccinated populations, older adults remain disproportionately affected by influenza, particularly during seasons characterized by antigenic mismatch. This highlights the need for tailored strategies, including enhanced vaccines and early antiviral intervention, specifically for this population [29]. Nevertheless, the per capita cost during this season was relatively lower, potentially reflecting the benefits of high vaccine coverage, reinforcing the importance of maintaining robust vaccination levels in older adults.

In contrast to the overall economic cost, per capita costs were highest in the 2018/19 season, followed by 2015/16. Both seasons experienced predominant circulation of influenza A/H1N1, consistent with subtype distributions in our data. Notably, during these periods, young adults with chronic conditions (20–64 years) exhibited a higher propensity for ICU admission, and in 2018/19, mortality among this group increased substantially, resulting in elevated direct and indirect costs, with per capita costs peaking. These findings align with growing evidence regarding the greater in-hospital severity of H1N1pdm infection compared to other subtypes [30,31]. Recent US analyses of over 100,000 hospitalized influenza patients found that while A/H3N2 accounted for more admissions, those infected with A/H1N1 had 1.4–1.7 times higher risks of ICU admission and mechanical ventilation across all ages, along with increased odds of death in young adults (18–64 years), particularly among unvaccinated individuals [30]. Age-related immunity gaps may partially explain this trend; older adults often retain cross-reactive antibodies from pre-2009 H1N1 exposure [32], whereas younger adults lack such immunity and typically have lower vaccination uptake, particularly among those with chronic conditions, as reflected by our findings. Our study revealed disproportionately low vaccination rates in confirmed cases, particularly among fatalities, during the H1N1 epidemic. These results emphasize the need for proactive influenza vaccination among young at-risk individuals, particularly during A/H1N1-predominant seasons.

Individuals with comorbidities, despite being a high-risk group for severe influenza, are underrepresented in the literature [2,5,8,12]. Consistent with previous studies, we observed higher healthcare costs in this population [2,8]. Among hospitalized patients, ICU admission rates ranged from 12.5% to 15.5%, with mortality rates of 4.0–6.7%, aligning with previous meta-analyses [33]. However, unlike earlier studies, our analysis offers greater granularity by stratifying the burden across multiple seasons, categorized by age and comorbidity type. Notably, among individuals with comorbidities, those with chronic kidney disease or immunocompromised conditions exhibited particularly high frequencies of influenza-related complications. While this may partly reflect their higher likelihood of seeking care at tertiary hospitals, the finding underscores the potential need for enhanced preventive strategies tailored to these groups. Moreover, the economic burden among young individuals with comorbidities was particularly pronounced during A/H1N1-dominant seasons. Further research is warranted to better understand subtype-specific risks among vulnerable subgroups and to guide strategies that reduce morbidity and mortality in those not yet covered by the National Immunization Program.

This study has several strengths and limitations. A key strength is the multi-center hospital-based surveillance with laboratory-confirmed cases over five seasons, providing detailed case-level data on outcomes and costs, in contrast to prior studies that relied on insurance claims or single-season data. However, certain limitations must also be addressed. First, only confirmed influenza cases were considered, potentially underestimating the disease burden, particularly incidence. Notably, claims data from a similar period in South Korea showed 6–15 times higher incidence rates than ours [21]. However, as data were collected through individual reviews, the outcome measures within confirmed cases were accurate and reliable. Moreover, despite conservative estimates, the disease burden remains significant. Second, although the HIMM surveillance system does not encompass all healthcare institutions nationwide, it comprises major tertiary hospitals across key regions in South Korea. While these hospitals are mostly situated in urban settings, this is reflective of Korea’s healthcare structure, where tertiary care centers serve as regional hubs even for rural populations due to the country’s small geographic size and well-connected healthcare referral system. Therefore, while the study may not fully capture local primary care settings, the network reflects national trends in the burden of influenza among adults. Third, given that only university hospitals were included, the study population may differ slightly from primary care clinics or secondary hospitals, potentially including a higher proportion of severe cases due to referral patterns. However, the threshold for visiting university hospitals, particularly emergency rooms, is relatively low in South Korea, and many patients use them as first-contact healthcare providers, similar to primary care settings. Fourth, although hospital-acquired influenza cases (symptom onset ≥ 48 h after admission) may involve more severe clinical courses and greater resource utilization than community-acquired cases [34], they accounted for a relatively small proportion of the study population (~7%). To avoid overestimation, cost calculations for these cases were conservatively limited to the standard 5-day antiviral treatment duration. Finally, incidence rates among individuals with comorbidities must be interpreted with caution. As nationwide data on comorbid individuals were unavailable, the proportion of at-risk individuals was estimated based on existing literature [35]. Variations in these estimates may significantly impact the calculated incidence rates.

In conclusion, this study provides comprehensive insights into the seasonal burden of influenza, considering the interplay of age, underlying health conditions, virus strains, and vaccination rates. It highlights the considerable economic burden in South Korea, noting variations across age groups and comorbidities by season. Strategies are needed to reduce morbidity and mortality in older adults and protect at-risk populations outside the National Immunization Program. These results could guide future research on the cost-effectiveness of influenza interventions and help optimize health policies and resource allocation. Further studies should evaluate the impact of seasonal influenza vaccine formulations and assess influenza burden in the post-COVID-19 era through prospective, population-based surveillance that includes both laboratory-confirmed and clinically suspected cases across diverse healthcare settings. These studies will help validate our estimates, capture broader disease dynamics, and assess the evolving burden amid changing virus ecology and vaccination strategies.

## Supporting information

S1 File This file contains supplementary tables (S1–S14) and supplementary figures (S1–S5). (DOCX)
