# Supplementary material for: Estimating the clinical and economic burden of medically attended influenza in South Korea, stratified by age and comorbidity: A five-season hospital-based surveillance data, 2014/15–2018/19
Source: PLoS One. 2025 Sep 17;20(9):e0317643. doi: 10.1371/journal.pone.0317643 (PMC12443274; doi:10.1371/journal.pone.0317643)
Supplement: S1 File — (DOCX) [file pone.0317643.s001.docx]

**Supporting information**

**S1 files.** This file includes Supplementary Methods, Supplementary Tables (S1–S14), and Supplementary Figures (S1–S5)

**Estimation of cost**

*Direct cost*

Direct costs refer to the expenses incurred through hospitalization or outpatient visits and can be subdivided into direct medical and non-medical costs. The direct medical cost was the sum of the expenses incurred for treatment at each hospital. For medications prescribed outside the hospital, the standard cost of the prescribed drug was calculated and included. To prevent overestimation, outpatient and inpatient costs included costs or medications related only to influenza. The calculation formula for direct non-medical costs (transportation and nursing costs) has been provided in the previous study.(1) Briefly, in this study, we multiplied the per capita transportation cost from the 'Report on the Korea Health Panel Survey of 2018'(2) by the number of hospitalization or outpatient visits in that year, with the assumption that one hospitalization corresponded to two visits (for admission and discharge). We calculated the daily nursing cost by multiplying the mode nursing expenses per day by the age-specific caregiving rate.(2) This daily nursing cost was then summated, taking into account the length of hospitalization.

*Indirect cost*

The formula for calculating indirect costs has been presented in a previous study.(1) The data used for the calculations in this study are detailed in S2 Table

**Table of contents**

**S1 Table.** Values for calculating the socioeconomic cost of five influenza seasons (2014/15–2018/19) in the Korean adult population

**S2 Table.** Daily salaries in Korea across each season

**S3 Table.** Catchment population for each season and age group

**S4 Table.** Baseline characteristics of the subpopulations among laboratory-confirmed influenza cases across the eight participating hospitals based on individuals with comorbidities

**S5 Table.** Baseline characteristics of laboratory-confirmed influenza cases across the eight participating hospitals stratified by age group

**S6 Table.** Baseline characteristics and clinical courses of patients with influenza-related deaths

**S7 Table.** Logistic regression analyses for factors associated with mortality among adult patients with laboratory-confirmed influenza

**S8 Table.** Medical resource utilization patterns in laboratory-confirmed influenza cases across eight participating hospitals stratified by age group

**S9 Table.** Medical resource utilization patterns of subpopulations of laboratory-confirmed influenza cases across eight participating hospitals based on individuals with comorbidities

**S10 Table.** Socioeconomic costs of five influenza seasons (2014/15–2018/19) among the Korean adult population stratified by age group

**S11 Table.** Socioeconomic costs of the five influenza seasons among Korean individuals with comorbidities stratified by age group

**S12 Table.** Socioeconomic costs of five influenza seasons (2014/15–2018/19) in Koreans with each comorbidity

**S13 Table.** Annual average cost per capita (socioeconomic cost) among total and at-risk individuals, assuming no productivity loss for the ≥ 65 age group

**S14 Table.** Data on influenza virus isolates by subtype in South Korea from the Korea Disease Control and Prevention Agency and World Health Organization vaccine composition during the 2014/15–2018/19 seasons

**S1 Fig.** Geographic distribution of participating hospitals across South Korea

**S2 Fig.** Estimated incidence (/100,000 persons) of laboratory-confirmed influenza and related morbidity and mortality during the study period among the total population (overall, 20–64, and ≥ 65 years), sub-population of at-risk individuals (overall, 20–64, and ≥ 65 years), and individuals with each comorbidity

**S3 Fig.** Annual average cost per capita (direct medical cost only), stratified by age and comorbidity

**S4 Fig.** Annual average cost per capita (socioeconomic cost), based on each comorbidity, assuming productivity loss occurs across all age groups

**S5 Fig.** Annual average cost per capita (socioeconomic cost), stratified by age and comorbidity, assuming no productivity loss for individuals aged 65 and older

**S1 Table. Values for calculating the socioeconomic cost of the five influenza seasons (2014/15–2018/19) in the Korean adult population**

|  | | | **2014/15** | **2015/16** | **2016/17** | **2017/18** | **2018/19** | **ref** |
| --- | --- | --- | --- | --- | --- | --- | --- | --- |
| Direct medical cost | | | Database of each participating hospitals | | | | |  |
| Direct non-medical costs | Transportation cost per visit* | | 22.44 | 22.64 | 24.07 | 21.44 | 20.71 | (2) |
|  | Nursing cost per day | | 63.64 | 63.64 | 72.73 | 72.73 | 81.82 | (2) |
|  | Caregiving rate | <60 years | 0.615 | 0.607 | 0.576 | 0.587 | 0.564 | (2) |
|  |  | ≥60 years | 0.721 | 0.728 | 0.704 | 0.717 | 0.726 |  |
| Indirect cost | | | Calculated based on age** (S2 table) | | | | |  |

* Inpatient cases were counted as two visits, considering both admission and discharge

** The age-specific monthly regular payment multiplied by the age-specific employment rate multiplied by the duration of productivity loss (for inpatients, the duration of hospitalization, and for outpatients, one-third of the outpatient visit days)

**S2 Table. Daily salaries in Korea across each season**

| Year | Age group | Monthly regular payment (USD) | Employment rate (%) | Daily regular payment (USD) |
| --- | --- | --- | --- | --- |
| 2014 | 20–24 years | 1,766.36 | 49.9 | 28.98 |
|  | 25–29 years | 2,308.18 | 75.3 | 57.14 |
|  | 30–34 years | 2,847.27 | 76.9 | 71.99 |
|  | 35–39 years | 3,253.64 | 76.0 | 81.30 |
|  | 40–44 years | 3,460.00 | 79.8 | 90.78 |
|  | 45–49 years | 3,436.36 | 82.0 | 92.64 |
|  | 50–54 years | 3,329.09 | 78.8 | 86.25 |
|  | 55–59 years | 2,914.55 | 72.6 | 69.57 |
|  | 60–64 years | 2,050.00 | 60.0 | 40.44 |
|  | ≥65 years | 2,050.00 | 31.8 | 21.43 |
| 2015 | 20–24 years | 1,713.64 | 51.6 | 26.06 |
|  | 25–29 years | 2,261.82 | 75.0 | 52.91 |
|  | 30–34 years | 2,779.09 | 77.8 | 65.84 |
|  | 35–39 years | 3,277.27 | 75.6 | 75.84 |
|  | 40–44 years | 3,540.91 | 79.9 | 85.45 |
|  | 45–49 years | 3,514.55 | 82.1 | 89.22 |
|  | 50–54 years | 3,402.73 | 79.4 | 84.17 |
|  | 55–59 years | 3,005.45 | 72.6 | 68.87 |
|  | 60–64 years | 2,120.91 | 61.2 | 40.33 |
|  | ≥65 years | 2,120.91 | 31.1 | 21.40 |
| 2016 | 20–24 years | 1,756.36 | 51.6 | 29.80 |
|  | 25–29 years | 2,331.82 | 76.6 | 58.72 |
|  | 30–34 years | 2,900.91 | 77.6 | 74.01 |
|  | 35–39 years | 3,341.82 | 76.6 | 84.16 |
|  | 40–44 years | 3,570.91 | 79.9 | 93.80 |
|  | 45–49 years | 3,601.82 | 82.0 | 97.10 |
|  | 50–54 years | 3,476.36 | 79.3 | 90.63 |
|  | 55–59 years | 3,095.45 | 72.8 | 74.09 |
|  | 60–64 years | 2,205.45 | 61.6 | 44.66 |
|  | ≥65 years | 2,205.45 | 31.3 | 22.70 |
| 2017 | 20–24 years | 1,789.09 | 50.6 | 29.76 |
|  | 25–29 years | 2,384.55 | 75.9 | 59.50 |
|  | 30–34 years | 2,944.55 | 77.9 | 75.41 |
|  | 35–39 years | 3,380.91 | 77.8 | 86.48 |
|  | 40–44 years | 3,618.18 | 79.4 | 94.45 |
|  | 45–49 years | 3,753.64 | 82.7 | 102.06 |
|  | 50–54 years | 3,688.18 | 79.8 | 96.76 |
|  | 55–59 years | 3,290.91 | 74.2 | 80.28 |
|  | 60–64 years | 2,270.91 | 62.5 | 46.66 |
|  | ≥65 years | 2,270.91 | 31.5 | 23.52 |
| 2018 | 20–24 years | 1,909.09 | 48.9 | 30.69 |
|  | 25–29 years | 2,497.27 | 76.9 | 63.14 |
|  | 30–34 years | 3,069.09 | 78.9 | 79.61 |
|  | 35–39 years | 3,520.91 | 77.8 | 90.06 |
|  | 40–44 years | 3,780.91 | 79.6 | 98.95 |
|  | 45–49 years | 3,886.36 | 82.2 | 105.03 |
|  | 50–54 years | 3,790.91 | 79.7 | 99.33 |
|  | 55–59 years | 3,417.27 | 74.7 | 83.92 |
|  | 60–64 years | 2,444.55 | 61.4 | 49.35 |
|  | ≥65 years | 2,444.55 | 32.2 | 25.88 |

*USD $1= KRW ₩1,100

Based on reconstituted Employment and Labor statistics (2013–2018)(3) and the economically active population.(4) The daily regular payment was calculated by multiplying the monthly regular payment by the employment rate for each age group and subsequently multiplying by 12/365.

**S3 Table. Catchment population for each season and age group.** The catchment population was calculated by multiplying the total population (stratified by age or comorbidity) by the ratio of annual patient visits of the eight participating hospitals to the nationwide annual patient visits.

| **Season** |  | **Total population^1)^** | **Sub-population: individuals with comorbidities^1)2)^** |
| --- | --- | --- | --- |
| 2014/15 | All adults (aged ≥20 years) | 2,056,164 | 838,915 |
|  | 20–49 years old | 823,146 | 352,806 |
|  | 50–64 years old | 667,385 |  |
|  | 64–75 years old | 300,614 | 447,448 |
|  | ≥75 years old | 243,938 |  |
| 2015/16 | All adults (aged ≥20 years) | 2,093,367 | 854,094 |
|  | 20–49 years old | 732,442 | 328,859 |
|  | 50–64 years old | 655,173 |  |
|  | 64–75 years old | 303,685 | 444,208 |
|  | ≥75 years old | 236,895 |  |
| 2016/17 | All adults (aged ≥20 years) | 2,212,431 | 902,672 |
|  | 20–49 years old | 745,947 | 343,023 |
|  | 50–64 years old | 702,719 |  |
|  | 64–75 years old | 322,095 | 481,186 |
|  | ≥75 years old | 263,317 |  |
| 2017/18 | All adults (aged ≥20 years) | 2,205,613 | 899,890 |
|  | 20–49 years old | 706,290 | 335,668 |
|  | 50–64 years old | 711,822 |  |
|  | 64–75 years old | 318,190 | 489,504 |
|  | ≥75 years old | 277,406 |  |
| 2018/19^3)^ | All adults (aged ≥20 years) | 2,193,490 | 894,944 |
|  | 20–49 years old | 734,033 | 342,765 |
|  | 50–64 years old | 732,409 |  |
|  | 64–75 years old | 330,517 | 515,377 |
|  | ≥75 years old | 298,932 |  |
| Individuals with certain comorbidities^4)^ | | | |
| 2014/15 | Diabetes mellitus |  | 339,267 |
|  | Chronic cardiovascular disease |  | 191,223 |
|  | Chronic pulmonary disease |  | 394,784 |
|  | Chronic renal disease |  | 65,797 |
|  | Immunocompromised |  | 94,584 |
| 2015/16 | Diabetes mellitus |  | 345,406 |
|  | Chronic cardiovascular disease |  | 194,683 |
|  | Chronic pulmonary disease |  | 401,926 |
|  | Chronic renal disease |  | 66,988 |
|  | Immunocompromised |  | 96,295 |
| 2016/17 | Diabetes mellitus |  | 365,051 |
|  | Chronic cardiovascular disease |  | 205,756 |
|  | Chronic pulmonary disease |  | 424,787 |
|  | Chronic renal disease |  | 70,798 |
|  | Immunocompromised |  | 101,772 |
| 2017/18 | Diabetes mellitus |  | 363,926 |
|  | Chronic cardiovascular disease |  | 205,122 |
|  | Chronic pulmonary disease |  | 423,478 |
|  | Chronic renal disease |  | 70,580 |
|  | Immunocompromised |  | 101,458 |
| 2018/19 | Diabetes mellitus |  | 361,926 |
|  | Chronic cardiovascular disease |  | 203,995 |
|  | Chronic pulmonary disease |  | 421,150 |
|  | Chronic renal disease |  | 70,192 |
|  | Immunocompromised |  | 100,901 |

*USD $1= KRW ₩1,100

^1)^ We calculated all adult (aged ≥20 years) catchment populations and the catchment population for each age group separately. Consequently, the sum of the catchment populations for each age group may not perfectly align with the total population of all age groups.

^2)^ Catchment populations of individuals with comorbidities were estimated using the same calculations as those for the general adult population. The proportion of individuals with comorbidities was assumed based on Korean reports. Based on a review of reports and literature in South Korea, the prevalence of individuals with one or more comorbidities among the overall adult population, those aged 20–64, and those aged 65 years and older was determined to be 40.8%, 23.5%, and 82.2%, respectively,.(5, 6) Owing to limitations in age-specific data, age groups were classified into two categories (20–64 and ≥65 years).

^3)^ For the 2018/19 season, we could not directly obtain data on the number of patients who visited the participating hospitals; therefore, we used the average of the other season values.

^4)^ Based on a previous study in South Korea, the prevalence rates applied were 16.5% for diabetes, 9.3% for chronic cardiovascular diseases (excluding hypertension), 19.2% for chronic pulmonary diseases, 3.2% for chronic kidney diseases, and 4.6% for immunodeficiency.(6) However, caution is needed when interpreting these values, as the cited paper estimated the number of individuals with comorbidities based on ICD-10 codes from the claims data. For instance, in the context of healthcare practices in South Korea, there is a tendency to overuse asthma codes (e.g., using diagnostic codes for nebulizer prescription claims), which could potentially result in an overestimation of the proportion of chronic pulmonary diseases.

**S4 Table. Baseline characteristics of the subpopulations among laboratory-confirmed influenza cases across the eight participating hospitals based on individuals with and without comorbidities**

|  | **2014/15** | **2015/16** | **2016/17** | **2017/18** | **2018/19** |
| --- | --- | --- | --- | --- | --- |
| **Without underlyng condition** |  |  |  |  |  |
| LCI cases, no. | 1518 | 1620 | 1255 | 2225 | 2187 |
| Symptom onset to seeking medical attention, median (IQR), days^1)^ | 1 (2) | 1 (2) | 1 (1) | 1 (2) | 1 (1) |
| Diagnosis method, no. (%) | | | | | |
| RAT | 1468 (96.7) | 1557 (96.1) | 1232 (98.2) | 2183 (98.1) | 2134 (97.6) |
| PCR | 112 (7.4) | 112 (6.9) | 69 (5.5) | 91 (4.1) | 85 (3.9) |
| Culture | 3 (0.2) | 2 (0.1) | 0 | 0 | 1 (<0.1) |
| Influenza subtype, no. (%) | | | | | |
| Influenza A/H3N2 | 37 (2.4) | 1 (0.1) | 24 (1.9) | 46 (2.1) | 9 (0.4) |
| Influenza A/H1N1 | 23 (1.5) | 18 (1.1) | 0 | 3 (0.1) | 53 (2.4) |
| Influenza A/subtype unknown | 982 (64.7) | 1286 (79.4) | 1160 (92.4) | 1288 (57.9) | 1720 (78.6) |
| Influenza B | 479 (31.6) | 321 (19.8) | 72 (5.7) | 891 (40.0) | 405 (18.5) |
| Both A & B | 3 (0.2) | 6 (0.4) | 1 (0.1) | 3 (0.1) | 0 |
| Age, median (Q1-Q3), years | 39 (31-53) | 37 (30-48) | 36 (28-49) | 40 (30-55) | 35 (27-46) |
| 20–49 years, no. (%) | 1059 (69.8) | 1238 (76.4) | 942 (75.1) | 1470 (66.1) | 1713 (78.3) |
| 50–64 years, no. (%) | 323 (21.3) | 289 (17.8) | 218 (17.4) | 516 (23.2) | 327 (15.0) |
| 65–74 years, no. (%) | 56 (3.7) | 54 (3.3) | 52 (4.1) | 134 (6.0) | 75 (3.4) |
| ≥75 years, no. (%) | 80 (5.3) | 39 (2.4) | 43 (3.4) | 105 (4.7) | 72 (3.3) |
| Male, no. (%) | 523 (34.5) | 573 (35.4) | 440 (35.1) | 826 (37.1) | 850 (38.9) |
| Influenza vaccination, no. (%)^2)^ | 300 (27.6) | 264 (19.4) | 201 (22.7) | 442 (27.2) | 335 (23.6) |
| 20–49 years, no. (%) | 171/757 (22.6) | 177/1049 (16.9) | 110/661 (16.6) | 209/1043 (20.0) | 182/1074 (16.9) |
| 50–64 years, no. (%) | 63/218 (28.9) | 35/228 (15.4) | 33/142 (23.2) | 73/361 (20.2) | 60/217 (27.6) |
| 65–74 years, no. (%) | 32/47 (68.1) | 32/48 (66.7) | 31/47 (66.0) | 88/123 (71.5) | 48/64 (75.0) |
| ≥75 years, no. (%) | 34/63 (54.0) | 20/33 (60.6) | 27/37 (73.0) | 72/97 (74.2) | 45/64 (70.3) |
| **Comorbidity ≥1** |  |  |  |  |  |
| LCI cases, no. | 1439 | 1032 | 1248 | 2642 | 1348 |
| Symptom onset to seeking medical attention, median (IQR), days^1)^ | 2 (3) | 2 (3) | 1 (3) | 1 (3) | 1 (3) |
| Diagnosis method, no. (%) | | | | | |
| RAT | 1240 (86.2) | 872 (84.5) | 1083 (86.8) | 2239 (84.7) | 1127 (83.6) |
| PCR | 395 (27.4) | 320 (31.0) | 364 (29.2) | 712 (26.9) | 365 (27.1) |
| Culture | 16 (1.1) | 2 (0.2) | 0 | 0 | 0 |
| Influenza subtype, no. (%) | | | | | |
| Influenza A/H3N2 | 48 (3.3) | 1 (0.1) | 51 (4.1) | 326 (12.3) | 41 (3.0) |
| Influenza A/H1N1 | 44 (3.1) | 34 (3.3) | 2 (0.2) | 16 (0.6) | 231 (17.1) |
| Influenza A/subtype unknown | 1002 (69.6) | 888 (86.0) | 1103 (88.4) | 1364 (51.6) | 968 (71.8) |
| Influenza B | 349 (24.3) | 120 (11.6) | 92 (7.4) | 954 (36.1) | 110 (8.2) |
| Both A & B | 4 (0.3) | 11 (1.1) | 3 (0.2) | 18 (0.7) | 2 (0.1) |
| Age, median (Q1-Q3), years | 70 (57-77) | 63 (53-75) | 69 (57-78) | 70 (59-79) | 66.5 (55-77) |
| 20–49 years, no. (%) | 203 (14.1) | 209 (20.3) | 175 (14.0) | 285 (10.8) | 246 (18.3) |
| 50–64 years, no. (%) | 347 (24.1) | 334 (32.4) | 343 (27.5) | 725 (27.4) | 375 (27.9) |
| 65–74 years, no. (%) | 380 (26.4) | 224 (21.7) | 295 (23.6) | 577 (21.8) | 308 (22.8) |
| ≥75 years, no. (%) | 509 (35.4) | 265 (25.7) | 435 (34.9) | 1055 (39.9) | 419 (31.1) |
| Male, no. (%) | 639 (44.4) | 464 (45.0) | 588 (47.1) | 1276 (48.3) | 671 (49.8) |
| Influenza vaccination, no. (%)^2)^ | 588 (55.4) | 382 (45.4) | 607 (56.4) | 1365 (59.9) | 490 (50.5) |
| 20–49 years, no. (%) | 39/157 (24.8) | 35/168 (20.8) | 26/124 (21.0) | 37/209 (17.7) | 22/155 (14.2) |
| 50–64 years, no. (%) | 90/243 (37.0) | 80/277 (28.9) | 78/251 (31.1) | 136/549 (24.8) | 69/234 (29.5) |
| 65–74 years, no. (%) | 193/282 (68.4) | 116/182 (63.7) | 188/282 (66.7) | 389/521 (74.7) | 161/246 (65.4) |
| ≥75 years, no. (%) | 266/379 (70.2) | 151/215 (70.2) | 315/419 (75.2) | 803/998 (80.5) | 241/335 (71.9) |
| **Diabetes mellitus** | | | | | |
| LCI cases, no. | 432 | 289 | 416 | 806 | 440 |
| Symptom onset to seeking medical attention, median (IQR), days^1)^ | 2 (2) | 2(4) | 1 (3) | 1 (2) | 1 (2) |
| Diagnosis method, no. (%) | |  |  |  |  |
| RAT | 378 (87.5) | 240 (83.0) | 350 (84.1) | 661 (82.0) | 349 (79.3) |
| PCR | 122 (28.2) | 106 (36.7) | 131 (31.5) | 246 (30.5) | 137 (31.1) |
| Culture | 7 (1.6) | 0 | 0 | 0 | 0 |
| Influenza subtype, no. (%) | |  |  |  |  |
| Influenza A/H3N2 | 18 (4.2) | 0 | 17 (4.1) | 114 (14.1) | 12 (2.7) |
| Influenza A/H1N1 | 17 (3.9) | 16 (5.5) | 0 | 7 (0.9) | 98 (22.3) |
| Influenza A/subtype unknown | 318 (73.6) | 240 (83.0) | 375 (90.1) | 428 (53.1) | 305 (69.3) |
| Influenza B | 81 (18.8) | 38 (13.1) | 24 (5.8) | 266 (33.0) | 26 (5.9) |
| Both A & B | 2 (0.5) | 5 (1.7) | 0 | 9 (1.1) | 1 (0.2) |
| Age, median (Q1-Q3), years | 72 (64-78) | 68 (58-77) | 72 (61-78) | 73 (63-79) | 69 (59-78) |
| 20–49 years, no. (%) | 24 (5.6) | 35 (12.1) | 38 (9.1) | 46 (5.7) | 40 (9.1) |
| 50–64 years, no. (%) | 94 (21.8) | 91 (31.5) | 98 (23.6) | 176 (21.8) | 131 (29.8) |
| 65–74 years, no. (%) | 143 (33.1) | 72 (24.9) | 114 (27.4) | 223 (27.7) | 116 (26.4) |
| ≥75 years, no. (%) | 171 (39.6) | 91 (31.5) | 166 (39.9) | 361 (44.8) | 153 (34.8) |
| Male, no. (%) | 206 (47.7) | 149 (51.6) | 197 (47.4) | 410 (50.9) | 239 (54.3) |
| Influenza vaccination, no. (%)^2)^ | 187 (57.9) | 103 (43.8) | 223 (59.0) | 483 (69.7) | 182 (56.0) |
| **Chronic cardiovascular disease** | | | | | |
| LCI cases, no. | 294 | 186 | 279 | 639 | 303 |
| Symptom onset to seeking medical attention, median (IQR), days^1)^ | 2 (3) | 1 (3) | 2 (3) | 1 (3) | 1 (3) |
| Diagnosis method, no. (%) | |  |  |  |  |
| RAT | 242 (82.3) | 147 (79.0) | 224 (80.3) | 540 (84.5) | 246 (81.2) |
| PCR | 100 (34.0) | 74 (39.8) | 98 (35.1) | 185 (29.0) | 90 (29.7) |
| culture | 1 (0.3) | 0 | 0 | 0 | 0 |
| Influenza subtype, no. (%) | |  |  |  |  |
| Influenza A/H3N2 | 9 (3.1) | 0 | 8 (2.9) | 87 (13.6) | 9 (3.0) |
| Influenza A/H1N1 | 9 (3.1) | 9 (4.8) | 0 | 4 (0.6) | 55 (18.2) |
| Influenza A/subtype unknown | 206 (70.1) | 162 (87.1) | 255 (91.4) | 308 (48.2) | 225 (74.3) |
| Influenza B | 71 (24.1) | 20 (10.8) | 16 (5.7) | 245 (38.3) | 15 (5.0) |
| Both A & B | 1 (0.3) | 5 (2.7) | 0 | 5 (0.8) | 1 (0.3) |
| Age, median (Q1-Q3), years | 74 (64-79) | 71 (61-79) | 75 (68-81) | 75 (65-81) | 73 (64-81) |
| 20–49 years, no. (%) | 16 (5.4) | 16 (8.6) | 13 (4.7) | 29 (4.5) | 14 (4.6) |
| 50–64 years, no. (%) | 59 (20.1) | 43 (23.1) | 44 (15.8) | 125 (19.6) | 68 (22.5) |
| 65–74 years, no. (%) | 82 (27.9) | 55 (29.6) | 82 (29.4) | 146 (22.8) | 84 (27.7) |
| ≥75 years, no. (%) | 137 (46.6) | 72 (38.7) | 140 (50.2) | 339 (53.1) | 137 (45.2) |
| Male, no. (%) | 145 (49.2) | 95 (51.1) | 129 (46.2) | 348 (54.5) | 162 (53.5) |
| Influenza vaccination, no. (%)^2)^ | 120 (58.3) | 84 (56.4) | 169 (65.5) | 384 (66.7) | 129 (54.9) |
| **Chronic pulmonary disease** | | | | | |
| LCI cases, no. | 312 | 199 | 239 | 515 | 224 |
| Symptom onset to seeking medical attention, median (IQR), days^1)^ | 2 (3) | 2 (3) | 2 (2) | 1 (3) | 2 (2) |
| Diagnosis method, no. (%) | |  |  |  |  |
| RAT | 239 (76.6) | 161 (80.9) | 186 (77.8) | 388 (75.3) | 168 (75.0) |
| PCR | 130 (41.7) | 74 (37.2) | 113 (47.3) | 228 (44.3) | 83 (37.1) |
| Culture | 6 (1.9) | 0 | 0 | 0 | 0 |
| Influenza subtype, no. (%) | |  |  |  |  |
| Influenza A/H3N2 | 13 (4.2) | 1 (0.5) | 7 (2.9) | 113 (21.9) | 7 (3.1) |
| Influenza A/H1N1 | 12 (3.8) | 8 (4.0) | 1 (0.4) | 2 (0.4) | 58 (25.9) |
| Influenza A/subtype unknown | 226 (72.4) | 164 (82.4) | 210 (87.9) | 212 (41.2) | 137 (61.2) |
| Influenza B | 61 (19.6) | 30 (15.1) | 22 (9.2) | 191 (37.1) | 23 (10.3) |
| Both A & B | 0 | 4 (2.0) | 1 (0.4) | 3 (0.6) | 1 (0.4) |
| Age, median (Q1-Q3), years | 71 (61.5-78) | 69 (59-78) | 72 (63-80) | 73 (64-79) | 69 (57.5-77) |
| 20–49 years, no. (%) | 29 (9.3) | 24 (12.1) | 20 (8.4) | 34 (6.6) | 36 (16.1) |
| 50–64 years, no. (%) | 66 (21.2) | 48 (24.1) | 50 (20.9) | 113 (21.9) | 48 (21.4) |
| 65–74 years, no. (%) | 101 (32.4) | 58 (29.1) | 68 (28.5) | 129 (25.0) | 62 (27.7) |
| ≥75 years, no. (%) | 116 (37.2) | 69 (34.7) | 101 (42.3) | 239 (46.4) | 78 (34.8) |
| Male, no. (%) | 145 (46.5) | 95 (47.7) | 128 (53.6) | 299 (58.1) | 119 (53.1) |
| Influenza vaccination, no. (%)^2)^ | 162 (66.7) | 87 (54.7) | 142 (65.7) | 340 (73.3) | 93 (53.8) |
| **Chronic renal disease** | | | | | |
| LCI cases, no. | 151 | 108 | 137 | 313 | 157 |
| Symptom onset to seeking medical attention, median (IQR), days^1)^ | 1 (3) | 1(3) | 2 (3) | 1 (2) | 1 (2) |
| Diagnosis method, no. (%) | |  |  |  |  |
| RAT | 132 (87.4) | 97 (89.8) | 116 (84.7) | 248 (79.2) | 128 (81.5) |
| PCR | 38 (25.2) | 30 (27.8) | 35 (25.5) | 114 (36.4) | 46 (29.3) |
| Culture | 1 (0.7) | 0 | 0 | 0 | 0 |
| Influenza subtype, no. (%) | |  |  |  |  |
| Influenza A/H3N2 | 1 (0.7) | 0 | 8 (5.8) | 54 (17.3) | 4 (2.5) |
| Influenza A/H1N1 | 3 (2.0) | 5 (4.6) | 0 | 2 (0.6) | 29 (18.5) |
| Influenza A/subtype unknown | 114 (75.5) | 91 (84.3) | 117 (85.4) | 150 (47.9) | 116 (73.9) |
| Influenza B | 33 (21.9) | 15 (13.9) | 12 (8.8) | 109 (34.8) | 8 (5.1) |
| Both A & B | 0 | 3 (2.8) | 0 | 2 (0.6) | 0 |
| Age, median (Q1-Q3), years | 69 (55-77) | 63.5 (55-74.5) | 71 (59-77) | 70 (59-78) | 65 (55-76) |
| 20–49 years, no. (%) | 29 (19.2) | 21 (19.4) | 19 (13.9) | 42 (13.4) | 28 (17.8) |
| 50–64 years, no. (%) | 36 (23.8) | 34 (31.5) | 31 (22.6) | 74 (23.6) | 48 (30.6) |
| 65–74 years, no. (%) | 39 (25.8) | 26 (24.1) | 36 (26.3) | 84 (26.8) | 37 (23.6) |
| ≥75 years, no. (%) | 47 (31.1) | 27 (25.0) | 51 (37.2) | 113 (36.1) | 43 (27.4) |
| Male, no. (%) | 72 (47.7) | 59 (54.6) | 76 (55.5) | 173 (55.3) | 87 (55.4) |
| Influenza vaccination, no. (%)^2)^ | 53 (49.1) | 45 (50.6) | 65 (52.8) | 172 (60.6) | 62 (51.2) |
| **Immunocompromised^3)^** | | | | | |
| LCI cases, no. | 261 | 229 | 287 | 620 | 351 |
| Symptom onset to seeking medical attention, median (IQR), days^1)^ | 1 (3) | 1 (3) | 1 (3) | 1 (3) | 1 (2) |
| Diagnosis method, no. (%) | |  |  |  |  |
| RAT | 217 (83.1) | 196 (85.6) | 257 (89.5) | 530 (85.5) | 287 (81.8) |
| PCR | 75 (28.7) | 76 (33.2) | 75 (26.1) | 162 (26.1) | 108 (30.8) |
| Culture | 5 (1.9) | 0 | 0 | 0 | 0 |
| Influenza subtype, no. (%) | |  |  |  |  |
| Influenza A/H3N2 | 10 (3.8) | 0 | 9 (3.1) | 75 (12.1) | 15 (4.3) |
| Influenza A/H1N1 | 5 (1.9) | 4 (1.7) | 0 | 3 (0.5) | 69 (19.7) |
| Influenza A/subtype unknown | 176 (67.4) | 201 (87.8) | 261 (90.9) | 320 (51.6) | 228 (65.0) |
| Influenza B | 70 (26.8) | 25 (10.9) | 18 (6.3) | 224 (36.1) | 39 (11.1) |
| Both A & B | 0 | 1 (0.4) | 1 (0.3) | 2 (0.3) | 0 |
| Age, median (Q1-Q3), years | 66 (55-76) | 61 (52-72) | 65 (55-76) | 66 (57-76) | 64 (52.5-76) |
| 20–49 years, no. (%) | 42 (16.1) | 46 (20.1) | 45 (15.7) | 76 (12.3) | 73 (20.8) |
| 50–64 years, no. (%) | 77 (29.5) | 89 (38.9) | 91 (31.7) | 221 (35.6) | 107 (30.5) |
| 65–74 years, no. (%) | 62 (23.8) | 51 (22.3) | 73 (25.4) | 137 (22.1) | 75 (21.4) |
| ≥75 years, no. (%) | 80 (30.7) | 43 (18.8) | 78 (27.2) | 186 (30.0) | 96 (27.4) |
| Male, no. (%) | 139 (53.3) | 116 (50.7) | 156 (54.4) | 319 (51.5) | 182 (51.9) |
| Influenza vaccination, no. (%)^2)^ | 97 (49.7) | 84 (47.5) | 120 (48.6) | 269 (52.6) | 135 (53.8) |

LCI, laboratory-confirmed influenza; no, number; IQR, interquartile range; RAT, rapid antigen test; PCR, polymerase chain reaction; SD, standard deviation

^1)^ The duration from symptom onset to initial hospital visit, excluding cases of in-hospital onset.

^2)^ Influenza vaccine recipients during a specific season, including only those who received the vaccine 14 days or more before the onset of infection. When calculating the ratio, individuals with unknown vaccination statuses were excluded.

^3)^ Solid organ cancer, hematologic malignancies, bone marrow/organ transplant recipients, HIV infection, and individuals undergoing immunosuppressive therapy.

**S5 Table. Baseline characteristics of laboratory-confirmed influenza cases across the eight participating hospitals stratified by age group**

|  | **2014/15** | **2015/16** | **2016/17** | **2017/18** | **2018/19** |
| --- | --- | --- | --- | --- | --- |
| **20–49 years** |  |  |  |  |  |
| LCI cases, no. | 1,262 | 1,447 | 1,117 | 1,755 | 1,959 |
| Symptom onset to seeking medical attention, median (Q1–Q3), days^1)^ | 1 (1–3) | 1 (1–3) | 1 (1–2) | 1 (0–2) | 1 (0–2) |
| Diagnosis method, no. (%) | | | | | |
| RAT | 1,232 (97.6) | 1,397 (96.5) | 1,094 (97.9) | 1,730 (98.6) | 1,914 (97.7) |
| PCR | 90 (7.1) | 100 (6.9) | 67 (6.0) | 63 (3.6) | 91 (4.6) |
| Culture | 4 (0.3) | 0 | 0 | 0 | 1 (0.1) |
| Influenza subtype, no. (%) | | | | | |
| Influenza A/H3N2 | 36 (2.9) | 1 (0.1) | 24 (2.1) | 33 (1.9) | 5 (0.3) |
| Influenza A/H1N1 | 23 (1.8) | 21 (1.5) | 0 | 2 (0.1) | 55 (2.8) |
| Influenza A/subtype unknown | 838 (66.4) | 1,104 (76.3) | 1,031 (92.3) | 1,054 (60.1) | 1472 (75.1) |
| Influenza B | 367 (29.1) | 327 (22.6) | 64 (5.7) | 671 (38.2) | 427 (21.8) |
| Both A & B | 2 (0.2) | 6 (0.4) | 2 (0.2) | 5 (0.3) | 0 |
| Comorbidities, no. (%) | 203 (16.1) | 209 (14.4) | 175 (15.7) | 285 (16.2) | 246 (12.6) |
| Male, no. (%) | 456 (36.1) | 535 (37.0) | 419 (37.5) | 684 (39.0) | 783 (40.0) |
| Influenza vaccination, no. (%)^2)^ | 210/914 (23.0) | 212/1217 (17.4) | 136/785 (17.3) | 246/1252 (19.6) | 204/1229 (16.6) |
| **50–64 years** | | | | | |
| LCI cases, no. | 670 | 623 | 561 | 1,241 | 702 |
| Symptom onset to seeking medical attention, median (Q1–Q3), days^1)^ | 2 (1–3) | 2 (1–3) | 1 (0–3) | 1 (0–3) | 1 (1–2) |
| Diagnosis method, no. (%) | |  |  |  |  |
| RAT | 617 (92.1) | 560 (89.9) | 517 (92.2) | 1,137 (91.6) | 625 (89.0) |
| PCR | 107 (16.0) | 124 (19.9) | 94 (16.8) | 171 (13.8) | 115 (16.4) |
| Culture | 6 (0.9) | 2 (0.3) | 0 | 0 | 0 |
| Influenza subtype, no. (%) | |  |  |  |  |
| Influenza A/H3N2 | 20 (3.0) | 0 | 17 (3.0) | 69 (5.6) | 7 (1.0) |
| Influenza A/H1N1 | 15 (2.2) | 9 (1.4) | 2 (0.4) | 2 (0.2) | 81 (11.5) |
| Influenza A/subtype unknown | 389 (58.1) | 573 (92.0) | 507 (90.4) | 677 (54.6) | 572 (81.5) |
| Influenza B | 248 (37.0) | 43 (6.9) | 35 (6.2) | 495 (39.9) | 42 (6.0) |
| Both A & B | 2 (0.3) | 2 (0.3) | 0 | 2 (0.2) | 0 |
| Comorbidities, no. (%) | 347 (51.8) | 334 (53.6) | 343 (61.1) | 725 (58.4) | 375 (53.4) |
| Male, no. (%) | 240 (35.8) | 236 (37.9) | 221 (39.4) | 528 (42.5) | 309 (44.0) |
| Influenza vaccination, no. (%)^2)^ | 153/461 (33.2) | 115/505 (22.8) | 111/393 (28.2) | 209/910 (23.0) | 126/451 (27.9) |
| **65–74 years** | | | | | |
| LCI cases, no. | 436 | 278 | 347 | 711 | 383 |
| symptom onset to seeking medical attention, median (Q1–Q3), days^1)^ | 2 (0–3) | 2 (0–3) | 1 (0–3) | 1 (0–3) | 1 (0–3) |
| Diagnosis method, no. (%) | |  |  |  |  |
| RAT | 372 (85.2) | 223 (80.2) | 303 (87.3) | 609 (85.7) | 323 (84.3) |
| PCR | 124 (28.4) | 89 (32.0) | 101 (29.1) | 184 (25.9) | 105 (27.4) |
| culture | 2 (0.5) | 1 (0.4) | 0 | 0 | 0 |
| Influenza subtype, no. (%) | |  |  |  |  |
| Influenza A/H3N2 | 15 (3.4) | 1 (0.4) | 17 (4.9) | 95 (13.4) | 10 (2.6) |
| Influenza A/H1N1 | 13 (3.0) | 10 (3.6) | 0 | 3 (0.4) | 64 (16.7) |
| Influenza A/subtype unknown | 312 (71.6) | 228 (82.0) | 306 (88.2) | 352 (49.5) | 283 (73.9) |
| Influenza B | 96 (22.0) | 42 (15.1) | 24 (6.9) | 268 (37.0) | 27 (7.0) |
| Both A & B | 0 | 3 (1.1) | 1 (0.3) | 7 (1.0) | 1 (0.3) |
| Comorbidities, no. (%) | 380 (87.2) | 224 (80.6) | 295 (85.0) | 577 (81.2) | 308 (80.4) |
| Male, no. (%) | 200 (45.9) | 139 (50.0) | 170 (49.0) | 345 (48.5) | 203 (53.0) |
| Influenza vaccination, no. (%)^2)^ | 225/329 (68.4) | 148/230 (64.3) | 219/329 (66.6) | 477/644 (74.1) | 209/310 (67.4) |
| **≥75 years** | | | | | |
| LCI cases, no. | 589 | 304 | 478 | 1,160 | 491 |
| Symptom onset to seeking medical attention, median (Q1–Q3), days^1)^ | 2 (0–3) | 1 (0–3) | 1 (0–3) | 1 (0–3) | 1 (0–3) |
| Diagnosis method, no. (%) | |  |  |  |  |
| RAT | 487 (82.7) | 249 (81.9) | 401 (83.9) | 946 (81.6) | 399 (81.3) |
| PCR | 186 (31.6) | 119 (39.1) | 171 (35.8) | 385 (33.2) | 139 (28.3) |
| Culture | 7 (1.2) | 1 (0.3) | 0 | 0 | 0 |
| Influenza subtype, no. (%) | |  |  |  |  |
| Influenza A/H3N2 | 14 (2.4) | 0 | 17 (3.6) | 175 (15.1) | 28 (5.7) |
| Influenza A/H1N1 | 16 (2.7) | 12 (3.9) | 0 | 12 (1.0) | 84 (17.1) |
| Influenza A/subtype unknown | 445 (75.6) | 269 (88.5) | 419 (87.7) | 569 (49.1) | 361 (73.5) |
| Influenza B | 117 (19.9) | 29 (9.5) | 41 (8.6) | 411 (35.4) | 19 (3.9) |
| Both A & B | 3 (0.5) | 6 (2.0) | 1 (0.2) | 7 (0.6) | 1 (0.2) |
| Comorbidities, no. (%) | 509 (86.4) | 265 (87.2) | 435 (91.0) | 1,055 (90.9) | 419 (85.3) |
| Male, no. (%) | 266 (45.2) | 127 (41.8) | 218 (45.6) | 545 (47.0) | 226 (46.0) |
| Influenza vaccination, no. (%)^2)^ | 300/442 (67.9) | 171/248 (69.0) | 342/456 (75.0) | 875/1095 (80.0) | 286/399 (71.7) |

LCI, laboratory-confirmed influenza; no, number; IQR, interquartile range; RAT, rapid antigen test; PCR, polymerase chain reaction; SD, standard deviation

^1)^ The duration from symptom onset to initial hospital visit, excluding cases of in-hospital onset.

^2)^ Influenza vaccine recipients during a specific season, including only those who received the vaccine 14 days or more before the onset of infection. When calculating the ratio, individuals with unknown vaccination statuses were excluded.

**S6 Table. Baseline characteristics and clinical courses of patients with influenza-related deaths**

|  | **2014/15**  **(n=50)** | **2015/16**  **(n=32)** | **2016/17**  **(n=30)** | **2017/18**  **(n=80)** | **2018/19**  **(n=47)** |
| --- | --- | --- | --- | --- | --- |
| Symptom onset to seeking medical attention, median (IQR), days^1)^ | 2 (0–4) | 2 (0–4) | 2 (0–5) | 2 (0–3) | 1 (0–3) |
| Diagnosis method, no. (%) | | | | | |
| RAT | 36 (72.0) | 25 (78.1) | 13 (43.3) | 52 (65.0) | 32 (68.0) |
| PCR | 28 (56.0) | 15 (46.9) | 23 (76.7) | 52 (65.0) | 29 (61.7) |
| Culture | 1 (2.0) | 0 (0.0) | 0 (0.0) | 2 (2.5) | 0 (0.0) |
| Influenza subtype, no. (%) | | | | | |
| Influenza A/H3N2 | 3 (6.0) | 0 (0.0) | 2 (6.7) | 29 (36.3) | 4 (8.5) |
| Influenza A/H1N1 | 1 (2.0) | 0 (0.0) | 0 (0.0) | 2 (2.5) | 21 (44.7) |
| Influenza A/unknown | 34 (68.0) | 29 (90.6) | 26 (86.7) | 17 (21.3) | 18 (38.3) |
| Influenza B | 12 (24.0) | 3 (9.3) | 2 (6.7) | 31 (38.8) | 4 (8.5) |
| Both A & B | 1 (2.0) | 1 (3.1) | 1 (3.3) | 2 (2.5) | 0 (0.0) |
| Age, median (Q1-Q3), years | 77.5 (71-84) | 72.5 (59.5-79.5) | 71 (62-80) | 79 (69-85) | 73 (60.5-81) |
| 20–49 years, no. (%) | 0 (0.0) | 3 (9.4) | 2 (6.7) | 1 (1.3) | 4 (8.5) |
| 50–64 years, no. (%) | 2 (4.0) | 9 (28.1) | 6 (20.0) | 11 (13.8) | 10 (21.3) |
| 65–74 years, no. (%) | 16 (32.0) | 6 (18.8) | 11 (36.7) | 20 (25.0) | 13 (27.6) |
| ≥75 years, no. (%) | 32 (64.0) | 14 (43.8) | 11 (36.7) | 48 (60.0) | 20 (42.6) |
| Male, no. (%) | 34 (68.0) | 19 (59.4) | 21 (70.0) | 48 (36.3) | 27 (57.4) |
| Influenza vaccination, no. (%)^2)^ | 17/39 (43.6) | 8/24 (33.3) | 19/28 (67.9) | 45/66  (68.2) | 14/31 (45.2) |
| 20–49 years, no. (%) | 0/0 (0.0) | 0/2 (0.0) | 0/1 (0.0) | 0/0 (0.0) | 0/2 (0.0) |
| 50–64 years, no. (%) | 1/2 (50.0) | 2/7 (28.6) | 4/5 (80.0) | 2/6 (33.3) | 1/5 (20.0) |
| 65–74 years, no. (%) | 3/13 (23.1) | 1/5 (20.0) | 7/11 (63.6) | 13/16 (81.3) | 5/9 (55.6) |
| ≥75 years, no. (%) | 13/24 (54.2) | 5/10 (50.0) | 8/11 (72.7) | 30/44 (68.2) | 8/15 (53.3) |
| Comorbidities≥1, no. (%) | 49 (98.0) | 25 (78.1) | 24 (80.0) | 74 (92.5) | 46 (97.9) |
| Diabetes | 17 (34.0) | 3 (9.4) | 14 (46.7) | 27 (33.8) | 21 (44.7) |
| Hypertension | 35 (70.0) | 17 (53.1) | 16 (53.3) | 40 (50.0) | 29 (61.7) |
| Chronic cardiovascular disease (except hypertension) | 15 (30.0) | 7 (21.9) | 10 (33.3) | 20 (25.0) | 11 (23.4) |
| Chronic pulmonary disease | 10 (20.0) | 8 (25.0) | 4 (13.3) | 17 (21.3) | 6 (12.8) |
| Chronic renal disease | 6 (12.0) | 6 (18.8) | 6 (20.0) | 12 (15.0) | 4 (8.5) |
| Chronic liver disease | 2 (4.0) | 0 (0.0) | 2 (6.7) | 6 (6.3) | 4 (8.5) |
| Cerebrovascular disease | 15 (30.0) | 4 (2.5) | 6 (20.0) | 28 (35.0) | 15 (31.9) |
| Neuromuscular disease | 0 (0.0) | 1 (3.1) | 0 (0.0) | 4 (5.0) | 1 (2.1) |
| Autoimmune disease | 1 (2.0) | 4 (12.5) | 1 (3.3) | 2 (2.5) | 1 (2.1) |
| Immunocompromised^3)^ | 9 (18.0) | 7 (21.9) | 7 (23.3) | 19 (23.8) | 20 (42.6) |
| Pregnancy, no. (%) | 0 (0.0) | 0 (0.0) | 0 (0.0) | 0 (0.0) | 0 (0.0) |
| **Clinical course** | | | | | |
| ICU admission, no. (%) | 30 (60.0) | 15 (46.9) | 21 (70.0) | 38 (47.5) | 15 (31.9) |
| Median length of admission (Q1–Q3), days | 8 (5–14) | 13 (5–19.5) | 5 (5–15) | 6 (4–13) | 11 (5–19) |
| Median length of ICU stay (Q1–Q3), days | 4 (2–9) | 7 (2.8–11.5) | 9 (3–17) | 7 (3–12) | 7 (2–15) |
| Complication: total, no. (%) | 48 (96.0) | 28 (87.5) | 29 (96.7) | 74 (92.5) | 42 (89.4) |
| Pneumonia, no. (%) | 40 (80.0) | 22 (68.8) | 26 (86.7) | 62 (77.5) | 39 (83.0) |
| Encephalopathy, no. (%) | 1 (2.0) | 0 (0.0) | 0 (0.0) | 2 (2.5) | 0 (0.0) |
| Myocarditis, no. (%) | 1 (2.0) | 0 (0.0) | 1 (3.3) | 0 (0.0) | 1 (2.1) |
| AKI, no. (%) | 11 (22.0) | 2 (6.3) | 8 (26.7) | 15 (18.8) | 8 (17.0) |
| Rhabdomyolysis, no. (%) | 4 (8.0) | 0 (0.0) | 0 (0.0) | 1 (1.25) | 0 (0.0) |
| Antiviral agent use, no. (%) | 43 (86.0) | 31 (96.9) | 26 (86.7) | 71 (88.75) | 45 (95.7) |
| Oseltamivir | 7 (14.0) | 13 (40.6) | 5 (16.7) | 21 (26.25) | 13 (27.7) |
| Peramivir | 37 (74.0) | 19 (59.4) | 23 (76.7) | 53 (66.24) | 42 (89.4) |
| Zanamivir | 0 (0.0) | 0 (0.0) | 0 (0.0) | 0 (0.0) | 0 (0.0) |
| Co-infection with other viruses, no. (%) ** | 0/32 (0.0) | 3/15 (9.4) | 4/26 (13.3) | 10/56 (12.5) | 6/36 (16.7) |
| RSV | 0 (0.0) | 1 (3.1) | 1 (3.3) | 1 (1.3) | 1 (2.1) |
| HBoV | 0 (0.0) | 0 (0.0) | 0 (0.0) | 1 (1.3) | 2 (4.3) |
| HRV | 0 (0.0) | 0 (0.0) | 1 (3.3) | 1 (1.3) | 2 (4.3) |
| Coronavirus NL63 | 0 (0.0) | 2 (6.3) | 2 (6.7) | 5 (6.3) | 2 (4.3) |
| HEV | 0 (0.0) | 0 (0.0) | 0 (0.0) | 2 (2.5) | 0 (0.0) |

LCI, laboratory-confirmed influenza; no, number; IQR, interquartile range; RAT, rapid antigen test; PCR, polymerase chain reaction; SD, standard deviation; AKI, acute kidney injury; RSV, respiratory syncytial virus; HBoV, human bocavirus; HRV, human rhinovirus; HEV, hepatitis E virus.

^1)^ The duration from symptom onset to initial hospital visit, excluding cases of in-hospital onset

^2)^ Influenza vaccine recipients during a specific season, including only those who received the vaccine 14 days or more before the onset of infection. When calculating the ratio, individuals with unknown vaccination statuses were excluded

^3)^ Solid organ cancer, hematologic malignancies, bone marrow/organ transplant recipients, HIV infection, and individuals undergoing immunosuppressive therapy

^4)^ The calculation of the ratio excluded individuals who were not tested for concurrent infections with other respiratory viruses

**S7 Table. Logistic regression analyses for factors associated with mortality among adult patients with laboratory-confirmed influenza**

|  | category | Died, no. (%) | p-value | Adjusted OR (95% CI) | p-value |
| --- | --- | --- | --- | --- | --- |
| Time from symptom onset to medical attention (Days) | <3days | 124 (1.1%) | - | ref |  |
|  | ≥3 days | 85 (2.0%) | <0.001 | 1.456 (1.061-1.999) | 0.020 |
| Influenza A | No | 48 (1.3%) | - |  |  |
|  | Yes | 191 (1.5%) | 0.338 |  |  |
| Age | <65 years | 48 (0.4%) | - | ref |  |
|  | ≥65 years | 191 (3.7%) | <0.001 | 7.974 (4.762-13.354) | <0.001 |
| Influenza vaccination | No | 85 (1.1%) | - | ref |  |
|  | Yes | 103 (2.1%) | <0.001 | 0.571 (0.410-0.795) | 0.001 |
| Presence of comorbidity | No | 18 (0.2%) | - | ref |  |
|  | Yes | 221 (2.9%) | <0.001 | 4.933 (2.647-9.193) | <0.001 |
| Antiviral agent use | No | 23 (3.3%) | - | Ref |  |
|  | Yes | 216 (1.4%) | <0.001 | 0.711 (0.396-1.276) | 0.253 |
| Viral coinfection^1)^ | No | 142 (4.3%) | - |  |  |
|  | Yes | 23 (10.0%) | <0.001 | NA^2)^ | NA |

^1)^ Viral coinfection was defined as detection of one or more non-influenza respiratory viruses (e.g., respiratory syncytial virus, human bocavirus, human rhinovirus) bye multiplex PCR

^2)^ Variables with p-values <0.1 in the univariable analysis were included in the multivariable logistic regression model, except for viral coinfection, which were excluded due to high rates of missing data (approximately 80%,)

**S8 Table. Medical resource utilization patterns in laboratory-confirmed influenza cases across the eight participating hospitals stratified by age group**

|  |  | **2014/15** | **2015/16** | **2016/17** | **2017/18** | **2018/19** |
| --- | --- | --- | --- | --- | --- | --- |
| **Hospital admission, no. (%)** | **Overall** | 900 (30.4) | 743 (28.0) | 808 (32.3) | 1693 (34.8) | 908 (25.7) |
|  | **20–49 years** | 145 (11.5) | 164 (11.3) | 128 (11.5) | 183 (10.4) | 167 (8.5) |
|  | **50–64 years** | 185 (27.6) | 216 (34.7) | 186 (33.2) | 395 (31.8) | 225 (32.1) |
|  | **65–74 years** | 227 (52.1) | 150 (54.0) | 193 (55.6) | 354 (49.8) | 205 (53.5) |
|  | **≥75 years** | 343 (58.2) | 213 (70.1) | 301 (63.0) | 761 (65.6) | 311 (63.3) |
|  | **p-value** | <0.001 | <0.001 | <0.001 | <0.001 | <0.001 |
| **ICU admission, no. (%)** | **Overall** | 98 (3.3) | 102 (3.8) | 92 (3.7) | 202 (4.2) | 113 (3.2) |
|  | **20–49 years** | 7 (0.6) | 13 (0.9) | 7 (0.6) | 9 (0.5) | 16 (0.8) |
|  | **50–64 years** | 8 (1.2) | 28 (4.5) | 16 (2.9) | 44 (3.5) | 30 (4.3) |
|  | **65–74 years** | 28 (6.4) | 23 (8.3) | 24 (6.9) | 36 (5.1) | 27 (7.0) |
|  | **≥75 years** | 55 (9.3) | 38 (12.5) | 45 (9.4) | 113 (9.7) | 40 (8.1) |
|  | **p-value** | <0.001 | <0.001 | <0.001 | <0.001 | <0.001 |
| **Death, no. (%)** | **Overall** | 50 (1.7) | 32 (1.2) | 30 (1.2) | 80 (1.6) | 47 (1.3) |
|  | **20–49 years** | 0 | 3 (0.2) | 2 (0.2) | 1 (0.1) | 4 (0.2) |
|  | **50–64 years** | 2 (0.3) | 9 (1.4) | 6 (1.1) | 11 (0.9) | 10 (1.4) |
|  | **65–74 years** | 16 (3.7) | 6 (2.2) | 11 (3.2) | 20 (2.8) | 13 (3.4) |
|  | **≥75 years** | 32 (5.4) | 14(4.6) | 11 (2.3) | 48 (4.1) | 20 (4.1) |
|  | **p-value** | <0.001 | <0.001 | <0.001 | <0.001 | <0.001 |
| **Median frequency of clinic visit (Q1–Q3)** | **Overall** | 1 (1–1) | 1 (1–1) | 1 (1–1) | 1 (0–1) | 1 (1–1) |
|  | **20–49 years** | 1 (1–1) ^a^ | 1 (1–1) ^a^ | 1 (1–1) ^a^ | 1 (1–1) ^a^ | 1 (1–1) ^a^ |
|  | **50–64 years** | 1 (1–2) ^a^ | 1 (1–1) ^a,b^ | 1 (1–1) ^a^ | 1 (0–1) ^b^ | 1 (0–1) ^b^ |
|  | **65–74 years** | 1 (0–1) ^b^ | 1 (0–1) ^b^ | 1 (0–1) ^b^ | 1 (0–1) ^b^ | 1 (0–1) ^c^ |
|  | **≥75 years** | 1 (0–1) ^b^ | 0 (0–1) ^c^ | 1 (0–1) ^b^ | 0 (0–1) ^c^ | 1 (0–1) ^d^ |
|  | **p-value** | <0.001 | <0.001 | <0.001 | <0.001 | <0.001 |
| **Median length of admission (Q1–Q3), days** | **Overall** | 6 (5–10) | 6 (5–10) | 5 (5–8) | 5 (5–8) | 5 (5–9) |
|  | **20–49 years** | 5 (3–7)^a^ | 5 (4–8) ^a^ | 5 (4–5) ^a^ | 5 (4–5) ^a^ | 5 (4–7) |
|  | **50–64 years** | 5 (4–8)^a,b^ | 6 (5–10) ^a,b^ | 5 (5–6) ^a^ | 5 (5–7) ^b^ | 5 (5–9) |
|  | **65–74 years** | 7 (5–11)^b,c^ | 8 (5–11) ^a,b^ | 5 (5–8) ^a, b^ | 5 (5–9) ^b^ | 5 (5–9) |
|  | **≥75 years** | 8 (5–11)^c^ | 7 (5–13) ^b^ | 6 (5–11) ^b^ | 6 (5–10) ^b^ | 6 (5–9) |
|  | **p-value** | <0.001 | 0.007 | 0.011 | <0.001 | 0.107 |
| **Median length of ICU stay (Q1–Q3), days** | **Overall** | 5 (3–10) | 7 (4–13) | 7 (3–14) | 6 (4–10) | 6 (3–14) |
|  | **20–49 years** | 5 (3–13) | 13 (4–21) | 14 (11–21) | 7 (4–9) | 6 (4–10) |
|  | **50–64 years** | 3 (3–10) | 7 (4–12) | 11 (4–15) | 5 (4–13) | 8 (6–19) |
|  | **65–74 years** | 4 (2–10) | 6 (4–8) | 5 (3–13) | 8 (5–9) | 7 (4–13) |
|  | **≥75 years** | 5 (4–9) | 8 (3–11) | 7 (4–14) | 6 (3–10) | 5 (3–13) |
|  | **p-value** | 0.860 | 0.472 | 0.731 | 0.649 | 0.404 |
| **Complication: total, no. (%)** | **Overall** | 461 (15.6) | 334 (12.6) | 314 (12.5) | 699 (14.4) | 415 (11.7) |
|  | **20–49 years** | 37 (2.9) | 54 (3.7) | 21 (1.9) | 36 (2.1) | 55 (2.8) |
|  | **50–64 years** | 72 (10.7) | 83 (13.3) | 63 (11.2) | 115 (9.3) | 98 (14.0) |
|  | **65–74 years** | 121 (27.8) | 78 (28.1) | 79 (22.8) | 180 (25.3) | 106 (27.7) |
|  | **≥75 years** | 231 (39.2) | 119 (39.1) | 151 (31.6) | 368 (31.7) | 156 (31.8) |
|  | **p-value** | <0.001 | <0.001 | <0.001 | <0.001 | <0.001 |
| **Complication: pneumonia, no. (%)** | **Overall** | 360 (12.2) | 284 (10.7) | 245 (9.8) | 560 (11.5) | 352 (10.0) |
|  | **20–49 years** | 31 (2.5) | 49 (3.4) | 15 (1.3) | 28 (1.6) | 46 (2.3) |
|  | **50–64 years** | 53 (7.9) | 74 (11.9) | 49 (8.7) | 89 (7.2) | 84 (12.0) |
|  | **65–74 years** | 88 (20.2) | 63 (22.7) | 59 (17.0) | 138 (19.4) | 91 (23.8) |
|  | **≥75 years** | 188 (31.9) | 98 (32.2) | 122 (25.5) | 305 (26.3) | 131 (26.7) |
|  | **p-value** | <0.001 | <0.001 | <0.001 | <0.001 | <0.001 |
| **Antiviral agent use, no. (%)** | **Overall** | 2,727 (92.2) | 2,553 (96.3) | 2,370 (94.7) | 4,712 (96.8) | 3,464 (98.0) |
|  | **20–49 years** | 1177 (93.3) | 1409 (97.4) | 1068 (95.6) | 1718 (97.9) | 1933 (98.7) |
|  | **50–64 years** | 618 (92.2) | 593 (95.2) | 532 (94.8) | 1207 (97.3) | 685 (97.6) |
|  | **65–74 years** | 404 (92.7) | 264 (95.0) | 325 (93.7) | 682 (95.9) | 372 (97.1) |
|  | **≥75 years** | 528 (89.6) | 287 (94.4) | 445 (93.1) | 1105 (95.3) | 474 (96.5) |
|  | **p-value** | 0.014 | 0.002 | 0.025 | <0.001 | 0.001 |

P-values are from linear-by-linear association tests for ordinal categorical variables and one-way ANOVA with Scheffé’s post hoc test for continuous variables, comparing age groups within each season. The same letters indicate non-significant differences between groups based on Scheffé’s multiple comparison.

**S9 Table. Medical resource utilization patterns of subpopulations of laboratory-confirmed influenza cases across the eight participating hospitals based on individuals with comorbidities**

|  |  |  | **2014/15** | **2015/16** | **2016/17** | **2017/18** | **2018/19** |
| --- | --- | --- | --- | --- | --- | --- | --- |
| **Hospital admission, no. (%)** | **Without comorbidity** | **Overall** | 178 (11.7) | 170 (10.5) | 126 (10.0) | 221 (9.9) | 148 (6.8) |
|  |  | **20–49 years** | 88 (8.3) | 85 (6.9) | 62 (6.6) | 99 (6.7) | 85 (5.0) |
|  |  | **50–64 years** | 42 (13.0) | 51 (17.6) | 33 (15.1) | 61 (11.8) | 33 (10.1) |
|  |  | **65–74 years** | 16 (28.6) | 17 (31.5) | 16 (30.8) | 28 (20.9) | 13 (17.3) |
|  |  | **≥75 years** | 32 (40.0) | 17 (43.6) | 15 (34.9) | 33 (31.4) | 17 (23.6) |
|  |  | **p-value** | <0.001 | <0.001 | <0.001 | <0.001 | <0.001 |
|  | **Comorbidity ≥1** | **Overall** | 722 (50.2)*** | 573 (55.5)*** | 682 (54.6)*** | 1472 (55.7) *** | 760 (56.4)*** |
|  |  | **20–49 years** | 57 (28.1)*** | 79 (37.8)*** | 66 (37.7)*** | 84 (29.5)*** | 82 (33.3)*** |
|  |  | **50–64 years** | 143 (41.2)*** | 165 (49.4)*** | 153 (44.6)*** | 334 (46.1)*** | 192 (51.2)*** |
|  |  | **65–74 years** | 211 (55.5)*** | 133 (59.4)*** | 177 (60.0)*** | 326 (56.5)*** | 192 (62.3)*** |
|  |  | **≥75 years** | 311 (61.1)*** | 196 (74.0)*** | 286 (65.7)*** | 728 (69.0)*** | 294 (70.2)*** |
|  |  | **p-value** | <0.001 | <0.001 | <0.001 | <0.001 | <0.001 |
|  | **Diabetes mellitus** | | 223 (51.6) | 184 (63.7) | 250 (60.1) | 515 (63.9) | 293 (66.6) |
|  | **Chronic cardiovascular disease** | | 180 (61.2) | 121 (65.1) | 176 (63.1) | 396 (62.0) | 200 (66.0) |
|  | **Chronic pulmonary disease** | | 211 (67.6) | 136 (68.3) | 161 (67.4) | 338 (65.6) | 147 (65.6) |
|  | **Chronic renal disease** | | 84 (55.6) | 76 (70.4) | 81 (70.4) | 201 (64.2) | 99 (63.1) |
|  | **Immunocompromised** | | 156 (59.8) | 144 (62.9) | 181 (63.1) | 398 (64.2) | 214 (61.0) |
| **ICU admission, no. (%)** | **Without comorbidity** | **Overall** | 6 (0.4) | 13 (0.8) | 7 (0.6) | 16 (0.7) | 13 (0.6) |
|  |  | **20–49 years** | 3 (0.3) | 4 (0.3) | 2 (0.2) | 6 (0.4) | 8 (0.5) |
|  |  | **50–64 years** | 0 | 7 (2.4) | 1 (0.5) | 5 (1.0) | 3 (0.9) |
|  |  | **65–74 years** | 0 | 1 (1.9) | 2 (3.8) | 1 (0.7) | 1 (1.3) |
|  |  | **≥75 years** | 3 (3.8) | 1 (2.6) | 2 (4.7) | 4 (3.8) | 1 (1.4) |
|  |  | **p-value** | 0.001 | 0.001 | <0.001 | 0.001 | 0.115 |
|  | **Comorbidity ≥1** | **Overall** | 92 (6.4)*** | 89 (8.6)*** | 85 (6.8)*** | 186 (7.0)*** | 100 (7.4)*** |
|  |  | **20–49 years** | 4 (2.0)* | 9 (4.3)*** | 5 (2.9)** | 3 (1.1) | 8 (3.3)*** |
|  |  | **50–64 years** | 8 (2.3)** | 21 (6.3)* | 15 (4.4)** | 39 (5.4)*** | 27 (7.2)*** |
|  |  | **65–74 years** | 28 (7.4)* | 22 (9.8) | 22 (7.5) | 35 (6.1)* | 26 (8.4)* |
|  |  | **≥75 years** | 52 (10.2) | 37 (14.0) | 43 (9.9) | 109 (10.3)* | 39 (9.3)* |
|  |  | **p-value** | <0.001 | <0.001 | <0.001 | <0.001 | 0.005 |
|  | **Diabetes mellitus** | | 31 (7.2) | 36 (12.5) | 45 (10.8) | 72 (8.9) | 49 (11.1) |
|  | **Chronic cardiovascular disease** | | 27 (9.2) | 20 (10.8) | 31 (11.1) | 66 (10.3) | 31 (10.2) |
|  | **Chronic pulmonary disease** | | 25 (8.0) | 27 (13.6) | 20 (8.4) | 41 (8.0) | 18 (8.0) |
|  | **Chronic renal disease** | | 14 (9.3) | 14 (13.0) | 18 (15.7) | 34 (10.9) | 17 (10.8) |
|  | **Immunocompromised** | | 17 (6.5) | 17 (7.4) | 15 (5.2) | 31 (5.0) | 24 (6.8) |
| **Death, no. (%)** | **Without comorbidity** | **Overall** | 1 (0.1) | 7 (0.4) | 3 (0.2) | 6 (0.3) | 1 (<0.1) |
|  |  | **20–49 years** | 0 | 2 (0.2) | 1 (0.1) | 1 (0.1) | 0 |
|  |  | **50–64 years** | 0 | 4 (1.4) | 1 (0.5) | 1 (0.2) | 1(0.3) |
|  |  | **65–74 years** | 0 | 0 | 1 (1.9) | 0 | 0 |
|  |  | **≥75 years** | 1 (1.2) | 1 (2.6) | 0 | 4 (3.8) | 0 |
|  |  | **p-value** | 0.001 | 0.006 | 0.123 | <0.001 | 0.325 |
|  | **Comorbidity ≥1** | **Overall** | 49 (3.4)*** | 25 (8.6)*** | 27 (2.2)*** | 74 (2.8)*** | 46 (3.4)*** |
|  |  | **20–49 years** | 0 | 1 (0.5) | 1 (0.6) | 0 | 4 (1.6)*** |
|  |  | **50–64 years** | 2 (0.6) | 5 (1.5) | 5 (1.5) | 10 (1.4)* | 9 (2.4)* |
|  |  | **65–74 years** | 16 (4.2) | 6 (2.7) | 10 (3.4) | 20 (3.5)* | 9 (2.9) |
|  |  | **≥75 years** | 31 (6.1) | 13 (4.9) | 11 (2.5) | 44 (4.2) | 20 (4.8) |
|  |  | **p-value** | <0.001 | 0.001 | 0.081 | <0.001 | 0.013 |
|  | **Diabetes mellitus** | | 17 (3.9) | 3 (1.0) | 14 (3.4) | 27 (3.3) | 21 (4.8) |
|  | **Chronic cardiovascular disease** | | 15 (5.1) | 7 (3.8) | 10 (3.6) | 20 (3.1) | 11 (3.6) |
|  | **Chronic pulmonary disease** | | 13 (4.2) | 10 (5.0) | 6 (2.5) | 21 (4.1) | 6 (2.7) |
|  | **Chronic renal disease** | | 6 (4.0) | 6 (5.6) | 6 (5.2) | 12 (3.8) | 4 (2.5) |
|  | **Immunocompromised** | | 10 (3.8) | 7 (3.1) | 7 (2.4) | 19 (3.1) | 20 (5.7) |
| **Median frequency of clinic visit (Q1–Q3)** | **Without comorbidity** | | 1 (1–1) | 1 (1–1) | 1 (1–1) | 1 (1–1) | 1 (1–1) |
|  | **Comorbidity ≥1** | | 1 (0–1)*** | 1 (0–1)*** | 1 (0–1)*** | 1 (0–1)*** | 1 (0–1)*** |
|  | **Diabetes mellitus** | | 1 (0–1) | 1 (0–1) | 1 (0–1) | 0 (0–1) | 0 (0–1) |
|  | **Chronic cardiovascular disease** | | 1 (0–1) | 1 (0–1) | 1 (0–1) | 1 (0–1) | 0 (0–1) |
|  | **Chronic pulmonary disease** | | 1 (0–1) | 1 (0–1) | 1 (0–1) | 1 (0–1) | 0 (0–1) |
|  | **Chronic renal disease** | | 1 (0–1) | 0 (0–1) | 0 (0–1) | 1 (0–1) | 0 (0–1) |
|  | **Immunocompromised** | | 1 (0–1) | 0 (0–1) | 1 (0–1) | 0 (0–1) | 0 (0–1) |
| **Median length of admission (Q1–Q3), days** | **Without comorbidity** | | 5 (3–7) | 5 (4–9) | 5 (3–6) | 5 (4–6) | 5 (4–7) |
|  | **Comorbidity ≥1** | | 7 (5–11)*** | 7 (5–11)* | 5 (5–8) | 5 (5–9)** | 5 (5–9) |
|  | **Diabetes mellitus** | | 7 (5–12) | 7 (5–11) | 5 (5–8) | 5 (5–8) | 5 (5–9) |
|  | **Chronic cardiovascular disease** | | 7 (5–12) | 8 (5–12) | 5 (5–11) | 6 (5–9) | 5 (5–9) |
|  | **Chronic pulmonary disease** | | 8 (5–12) | 8 (5–12) | 7 (5–13) | 7 (5–10) | 7 (5–10) |
|  | **Chronic renal disease** | | 8 (5–12) | 6 (5–11) | 6 (5–8) | 5 (5–8) | 5 (5–9) |
|  | **Immunocompromised** | | 5 (5–9) | 6 (5–8) | 5 (5–6) | 5 (5–7) | 5 (5–8) |
| **Median length of ICU stay (Q1–Q3), days** | **Without comorbidity** | | 8 (2–13) | 8 (3–20) | 12 (3–13) | 11 (4–22) | 5 (3–11) |
|  | **Comorbidity ≥1** | | 5 (3–9) | 7 (4–12) | 7 (4–15) | 6 (3.5–9) | 7 (3.5–15.5) |
|  | **Diabetes mellitus** | | 4 (3–10) | 7 (4–14) | 7 (3–14) | 6 (4–9) | 7 (5–14) |
|  | **Chronic cardiovascular disease** | | 5 (3–5) | 7 (3–10) | 9 (4–17) | 6 (3–9) | 6 (3–11) |
|  | **Chronic pulmonary disease** | | 4 (3–7) | 5 (3–7) | 12 (6–20) | 6 (5–10) | 6 (5–15) |
|  | **Chronic renal disease** | | 4 (2–7) | 4 (3–9) | 5 (4–9) | 5 (2–8) | 6 (3–11) |
|  | **Immunocompromised** | | 3 (2–5) | 4 (4–7) | 4 (3–5) | 6 (4–9) | 8 (5–18) |
| **Complication: total, no. (%)** | **Without comorbidity** | | 60 (4.0) | 64 (4.0) | 28 (2.2) | 59 (2.7) | 54 (2.5) |
|  | **Comorbidity ≥1** | | 401 (27.9)*** | 270 (26.2)*** | 286 (22.9)*** | 640 (24.2)*** | 361 (26.8)*** |
|  | **Diabetes mellitus** | | 127 (29.4) | 80 (27.7) | 107 (25.7) | 224 (27.8) | 142 (32.3) |
|  | **Chronic cardiovascular disease** | | 102 (34.7) | 64 (34.4) | 83 (29.7) | 188 (29.4) | 113 (37.3) |
|  | **Chronic pulmonary disease** | | 163 (52.2) | 94 (47.2) | 98 (41.0) | 237 (46.0) | 103 (46.0) |
|  | **Chronic renal disease** | | 47 (31.1) | 29 (26.9) | 36 (26.3) | 88 (28.1) | 43 (27.4) |
|  | **Immunocompromised** | | 68 (26.1) | 38 (16.6) | 50 (17.4) | 122 (19.7) | 79 (22.5) |
| **Complication: pneumonia, no. (%)** | **Without comorbidity** | | 51 (3.4) | 60 (3.7) | 24 (1.9) | 54 (2.4) | 49 (2.2) |
|  | **Comorbidity ≥1** | | 309 (21.5)*** | 224 (21.7)*** | 221 (17.7)*** | 506 (19.2)*** | 303 (22.5)*** |
|  | **Diabetes mellitus** | | 102 (23.6) | 74 (25.6) | 83 (20.0) | 181 (22.5) | 123 (28.0) |
|  | **Chronic cardiovascular disease** | | 79 (26.9) | 51 (27.4) | 62 (22.2) | 146 (22.8) | 89 (29.4) |
|  | **Chronic pulmonary disease** | | 98 (31.4) | 59 (29.6) | 60 (25.1) | 149 (28.9) | 71 (31.7) |
|  | **Chronic renal disease** | | 41 (27.2) | 26 (24.1) | 29 (21.2) | 77 (24.6) | 36 (22.9) |
|  | **Immunocompromised** | | 59 (22.6) | 27 (11.8) | 43 (15.0) | 102 (16.5) | 72 (20.5) |
| **Antiviral agent use, no. (%)** | **Without comorbidity** | | 1411 (93.0) | 1578 (97.4) | 1201 (95.8) | 2180 (98.0) | 2162 (98.9) |
|  | **Comorbidity ≥1** | | 1316 (91.5) | 975 (94.5)*** | 1169 (93.7)* | 2532 (95.8)*** | 1302 (96.6)*** |
|  | **Diabetes mellitus** | | 403 (93.3) | 278 (96.2) | 394 (94.7) | 765 (94.9) | 424 (96.4) |
|  | **Chronic cardiovascular disease** | | 269 (91.5) | 175 (94.1) | 264 (94.6) | 612 (95.8) | 293 (96.7) |
|  | **Chronic pulmonary disease** | | 282 (90.4) | 182 (91.5) | 225 (94.1) | 488 (94.8) | 214 (95.5) |
|  | **Chronic renal disease** | | 140 (92.7) | 103 (95.4) | 132 (96.4) | 299 (95.5) | 153 (97.5) |
|  | **Immunocompromised** | | 238 (91.2) | 213 (93.0) | 276 (96.2) | 604 (97.4) | 339 (96.6) |

P-values are from linear-by-linear trend tests comparing age groups within each season

Asterisks indicate statistical significance between individuals with and without underlying conditions within each age group and overall. Categorical variables were compared using two-sided chi-square or Fisher’s exact tests, and continuous variables were analyzed using independent t-tests. Significance levels are denoted as follows: **p* < 0.05; ***p* < 0.01; ****p* < 0.001. Asterisks are shown only in the group with comorbidity to denote the direction of the difference. Comparisons were not conducted between individual comorbidity groups (e.g., diabetes vs. no diabetes).

**S10 Table. Socioeconomic costs of five influenza seasons (2014/15–2018/19) among the Korean adult population stratified by age group**

|  | | | **Estimated cost (USD)^1)^** | | | | |
| --- | --- | --- | --- | --- | --- | --- | --- |
|  |  |  | **2014/15** | **2015/16** | **2016/17** | **2017/18** | **2018/19** |
| **All adults** | | | | | | | |
| Direct cost | Direct medical cost | | 67,112,736 | 78,195,427 | 64,014,397 | 138,677,621 | 112,304,571 |
|  | Direct medical cost (/person) | | 1,165 | 1,523 | 1,382 | 1,520 | 1,670 |
|  | Outpatient cost (/person) | | 286 | 288 | 313 | 330 | 339 |
|  | Inpatient cost (/person) | | 3,174 | 4,697 | 3,625 | 3,750 | 5,520 |
|  | Direct non-medical cost | Transportation cost | 8,009,350 | 7,355,029 | 7,264,539 | 12,140,249 | 7,134,344 |
|  |  | Nursing cost | 6,537,272 | 5,883,582 | 6,311,418 | 12,175,736 | 7,705,788 |
| Indirect cost | Due to visiting clinic or admission | Excluding productivity loss for ≥65 years | 4,136,059 | 5,142,709 | 3,209,686 | 6,529,995 | 5,643,715 |
|  | Due to early death |  | 2,572,443 | 44,738,215 | 26,475,226 | 29,770,333 | 58,756,076 |
|  | Due to visiting clinic or admission | Including productivity loss for ≥65 years | 6,483,709 | 6,764,922 | 5,406,510 | 10,719,714 | 7,890,605 |
|  | Due to early death |  | 70,116,036 | 90,619,393 | 72,723,051 | 142,343,280 | 138,796,420 |
| Total cost (excluding productivity loss for ≥65 years) | | | 88,367,860 | 141,314,961 | 107,275,265 | 199,293,934 | 191,544,494 |
| Total cost (including productivity loss for ≥65 years) | | | 158,259,104 | 188,818,353 | 156,719,915 | 316,056,600 | 273,831,727 |
| **20–49 years** | | |  |  |  |  |  |
| Direct cost | Direct medical cost | | 15,109,135 | 30,670,735 | 18,542,422 | 23,757,637 | 37,292,807 |
|  | Direct medical cost (/person) | | 424 | 672 | 540 | 420 | 620 |
|  | Outpatient cost (/person) | | 211 | 238 | 239 | 238 | 282 |
|  | Inpatient cost (/person) | | 2,063 | 4,066 | 2,863 | 1,982 | 4,250 |
|  | Direct non-medical cost | Transportation cost | 2,170,564 | 2,945,959 | 1,979,033 | 2,665,684 | 2,822,000 |
|  |  | Nursing cost | 1,038,788 | 1,454,636 | 935,724 | 1,370,050 | 1,592,301 |
| Indirect cost | Due to visiting clinic or admission | | 3,166,447 | 4,123,565 | 2,631,849 | 4,153,123 | 4,600,062 |
|  | Due to early death (excluding productivity loss for ≥65 years) | | 0 | 40,889,798 | 28,901,977 | 16,724,059 | 62,654,879 |
|  | Due to early death (including productivity loss for ≥65 years) | | 0 | 46,073,263 | 32,807,321 | 18,809,218 | 71,395,020 |
| Total cost (excluding productivity loss for ≥65 years) | | | 21,484,934 | 80,084,692 | 52,991,004 | 48,670,554 | 108,962,048 |
| Total cost (including productivity loss for ≥65 years) | | | 21,484,934 | 85,268,158 | 56,896,348 | 50,755,713 | 117,702,189 |
| **50–64 years** | | |  |  |  |  |  |
| Direct cost | Direct medical cost | | 9,127,339 | 21,001,174 | 10,812,574 | 29,459,381 | 24,073,620 |
|  | Direct medical cost (/person) | | 864 | 2,033 | 1,208 | 1,469 | 2,128 |
|  | Outpatient cost (/person) | | 275 | 338 | 297 | 317 | 381 |
|  | Inpatient cost (/person) | | 2,408 | 5,227 | 3,045 | 3,934 | 5,831 |
|  | Direct non-medical cost | Transportation cost | 1,194,204 | 1,756,033 | 1,133,954 | 2,383,496 | 1,446,164 |
|  |  | Nursing cost | 839,800 | 1,382,007 | 864,166 | 2,194,310 | 1,524,174 |
| Indirect cost | Due to visiting clinic or admission | | 1,578,833 | 2,237,882 | 1,402,486 | 3,548,390 | 2,366,417 |
|  | Due to early death (excluding productivity loss for ≥65 years) | | 2,081,536 | 16,830,202 | 7,837,884 | 17,284,747 | 16,885,350 |
|  | Due to early death (including productivity loss for ≥65 years) | | 5,297,360 | 31,016,848 | 18,366,755 | 37,439,872 | 36,647,598 |
| Total cost (excluding productivity loss for ≥65 years) | | | 14,821,711 | 43,207,298 | 22,051,065 | 54,870,324 | 46,295,725 |
| Total cost (including productivity loss for ≥65 years) | | | 18,037,535 | 57,393,944 | 32,579,935 | 75,025,449 | 66,057,973 |
| **65–74 years** | | |  |  |  |  |  |
| Direct cost | Direct medical cost | | 11,389,933 | 9,380,009 | 9,826,527 | 19,150,651 | 15,809,045 |
|  | Direct medical cost (/person)  Outpatient cost (/person)  Inpatient cost (/person) | | 2,070 | 2,636 | 2,308 | 2,128 | 3,279 |
|  |  |  | 383 | 466 | 428 | 445 | 524 |
|  |  |  | 3,623 | 4,488 | 3,809 | 3,824 | 5,672 |
|  | Direct non-medical cost | Transportation cost | 1,244,025 | 943,114 | 1,024,748 | 1,674,758 | 984,444 |
|  |  | Nursing cost | 1,161,569 | 887,626 | 993,034 | 1,825,823 | 1,295,649 |
| Indirect cost | Due to visiting clinic or admission | Including productivity loss for ≥65 years | 576,854 | 433,870 | 468,759 | 886,868 | 598,239 |
|  | Due to early death |  | 19,708,667 | 7,311,764 | 13,883,252 | 27,622,537 | 18,171,663 |
| Total cost (excluding productivity loss for ≥65 years) | | | 13,795,527 | 11,210,749 | 11,844,309 | 22,651,233 | 18,089,138 |
| Total cost (including productivity loss for ≥65 years) | | | 34,081,048 | 18,956,383 | 26,196,321 | 51,160,638 | 36,859,040 |
| **≥75 years** | | |  |  |  |  |  |
| Direct cost | Direct medical cost | | 14,652,983 | 12,019,972 | 14,805,360 | 36,413,948 | 20,946,885 |
|  | Direct medical cost (/person)  Outpatient cost (/person)  Inpatient cost (/person) | | 2,424 | 3,514 | 2,882 | 2,866 | 3,949 |
|  |  |  | 561 | 524 | 661 | 617 | 619 |
|  |  |  | 3,761 | 4,791 | 4,189 | 4,045 | 5,876 |
|  | Direct non-medical cost | Transportation cost | 1,640,624 | 1,204,156 | 1,865,772 | 3,122,794 | 1,244,085 |
|  |  | Nursing cost | 1,574,341 | 1,183,364 | 1,886,203 | 3,580,981 | 1,681,933 |
| Indirect cost | Due to visiting clinic or admission | Including productivity loss for ≥65 years | 767,368 | 561,668 | 865,025 | 1,680,212 | 776,471 |
|  | Due to early death |  | 17,454,340 | 8,769,291 | 6,819,102 | 27,498,097 | 13,534,294 |
| Total cost (excluding productivity loss for ≥65 years) | | | 17,867,947 | 14,407,492 | 18,557,334 | 43,117,724 | 23,872,903 |
| Total cost (including productivity loss for ≥65 years) | | | 36,089,655 | 23,738,450 | 26,241,461 | 72,296,033 | 38,183,669 |

^1)^ USD $1= KRW ₩1,100

**S11 Table. Socioeconomic costs of the five influenza seasons among Korean individuals with comorbidities stratified by age group**

|  | | | **Estimated cost (USD)*** | | | | |
| --- | --- | --- | --- | --- | --- | --- | --- |
|  |  |  | **2014/15** | **2015/16** | **2016/17** | **2017/18** | **2018/19** |
| **All adults** | | | | | | | |
| Direct cost | Direct medical cost | | 54,385,362 | 56,451,510 | 53,256,267 | 115,583,481 | 91,541,564 |
|  | Direct medical cost (/person)  Outpatient cost (/person)  Inpatient cost (/person) | | 1,940 | 2,826 | 2,306 | 2,333 | 3,570 |
|  |  |  | 405 | 393 | 441 | 459 | 512 |
|  |  |  | 3,464 | 4,775 | 3,853 | 3,823 | 5,935 |
|  | Direct non-medical cost | Transportation cost | 6,163,999 | 5,277,253 | 5,986,807 | 10,051,691 | 5,326,924 |
|  |  | Nursing cost | 5,594,837 | 4,792,879 | 5,714,974 | 10,970,841 | 6,725,169 |
| Indirect cost | Due to visiting clinic or admission | Excluding productivity loss for ≥65 years | 2,203,518 | 2,916,209 | 2,000,757 | 4,271,874 | 3,295,171 |
|  | Due to early death |  | 2,572,443 | 13,654,026 | 16,221,708 | 19,437,779 | 57,186,873 |
|  | Due to visiting clinic or admission | Including productivity loss for ≥65 years | 4,363,815 | 4,400,214 | 4,090,759 | 8,226,135 | 5,406,183 |
|  | Due to early death |  | 69,418,007 | 49,977,617 | 58,344,909 | 125,277,729 | 134,837,828 |
| Total cost (excluding productivity loss for ≥65 years) | | | 70,920,160 | 83,091,878 | 83,180,513 | 160,315,665 | 164,075,701 |
| Total cost (including productivity loss for ≥65 years) | | | 139,926,020 | 120,899,473 | 127,393,716 | 270,109,877 | 243,837,667 |
| **20–49 years** | | |  |  |  |  |  |
| Direct cost | Direct medical cost | | 4,927,895 | 10,264,163 | 8,727,854 | 7,908,520 | 14,778,312 |
|  | Direct medical cost (/person)  Outpatient cost (/person)  Inpatient cost (/person) | | 860 | 1,557 | 1,621 | 860 | 1,957 |
|  |  |  | 295 | 281 | 289 | 303 | 409 |
|  |  |  | 2,306 | 3,657 | 3,821 | 2,193 | 5,053 |
|  | Direct non-medical cost | Transportation cost | 683,807 | 1,005,310 | 731,768 | 876,806 | 870,948 |
|  |  | Nursing cost | 471,875 | 745,592 | 536,172 | 702,237 | 807,484 |
| Indirect cost | Due to visiting clinic or admission | | 1,125,340 | 1,675,421 | 1,199,458 | 1,630,384 | 1,686,578 |
|  | Due to early death (excluding productivity loss for ≥65 years) | | 0 | 9,151,497 | 18,467,108 | 0 | 62,654,879 |
|  | Due to early death (including productivity loss for ≥65 years) | | 0 | 11,416,567 | 20,097,472 | 0 | 71,395,020 |
| Total cost (excluding productivity loss for ≥65 years) | | | 7,208,917 | 22,841,984 | 29,662,360 | 11,117,946 | 80,798,201 |
| Total cost (including productivity loss for ≥65 years) | | | 7,208,917 | 25,107,054 | 31,292,724 | 11,117,946 | 89,538,342 |
| **50–64 years** | | |  |  |  |  |  |
| Direct cost | Direct medical cost | | 7,063,221 | 15,342,355 | 8,573,965 | 22,367,322 | 20,780,698 |
|  | Direct medical cost (/person)  Outpatient cost (/person)  Inpatient cost (/person) | | 1,291 | 2,770 | 1,567 | 1,909 | 3,438 |
|  |  |  | 320 | 373 | 337 | 348 | 448 |
|  |  |  | 2,676 | 5,226 | 3,093 | 3,736 | 6,288 |
|  | Direct non-medical cost | Transportation cost | 865,842 | 1,258,653 | 889,263 | 1,883,094 | 1,142,446 |
|  |  | Nursing cost | 683,081 | 1,052,358 | 733,594 | 1,858,539 | 1,315,067 |
| Indirect cost | Due to visiting clinic or admission | | 1,154,701 | 1,617,510 | 1,103,012 | 2,865,860 | 1,906,267 |
|  | Due to early death (excluding productivity loss for ≥65 years) | | 2,081,536 | 6,886,227 | 4,408,987 | 16,757,968 | 15,555,811 |
|  | Due to early death (including productivity loss for ≥65 years) | | 5,297,360 | 15,120,764 | 13,517,175 | 34,897,016 | 33,293,822 |
| Total cost (excluding productivity loss for ≥65 years) | | | 11,848,381 | 26,157,102 | 15,708,821 | 45,732,783 | 40,700,290 |
| Total cost (including productivity loss for ≥65 years) | | | 15,064,205 | 34,391,639 | 24,817,008 | 63,871,831 | 58,438,301 |
| **65–74 years** | | |  |  |  |  |  |
| Direct cost | Direct medical cost | | 10,770,393 | 8,448,375 | 9,087,866 | 17,855,602 | 14,864,314 |
|  | Direct medical cost (/person)  Outpatient cost (/person)  Inpatient cost (/person) | | 2,246 | 2,947 | 2,511 | 2,445 | 3,834 |
|  |  |  | 394 | 489 | 445 | 517 | 583 |
|  |  |  | 3,729 | 4,629 | 3,889 | 3,929 | 5,799 |
|  | Direct non-medical cost | Transportation cost | 1,155,368 | 833,591 | 931,428 | 1,536,620 | 900,257 |
|  |  | Nursing cost | 1,089,767 | 793,942 | 917,034 | 1,714,927 | 1,208,175 |
| Indirect cost | Due to visiting clinic or admission | Including productivity loss for ≥65 years | 537,453 | 384,923 | 428,203 | 819,676 | 550,467 |
|  | Due to early death |  | 19,708,667 | 7,311,764 | 12,485,598 | 27,622,537 | 18,171,663 |
| Total cost (excluding productivity loss for ≥65 years) | | | 13,015,528 | 10,075,908 | 10,936,327 | 21,107,149 | 16,972,746 |
| Total cost (including productivity loss for ≥65 years) | | | 33,261,649 | 17,772,595 | 23,850,128 | 49,549,362 | 35,694,876 |
| **≥75 years** | | |  |  |  |  |  |
| Direct cost | Direct medical cost | | 13,497,077 | 11,318,397 | 14,141,123 | 34,230,898 | 20,096,943 |
|  | Direct medical cost (/person)  Outpatient cost (/person)  Inpatient cost (/person) | | 2,584 | 3,796 | 3,025 | 2,962 | 4,440 |
|  |  |  | 584 | 526 | 683 | 642 | 675 |
|  |  |  | 3,858 | 4,947 | 4,245 | 4,005 | 6,041 |
|  | Direct non-medical cost | Transportation cost | 1,494,847 | 1,122,384 | 1,806,011 | 2,968,756 | 1,180,102 |
|  |  | Nursing cost | 1,444,401 | 1,108,296 | 1,833,930 | 3,427,347 | 1,616,478 |
| Indirect cost | Due to visiting clinic or admission | Including productivity loss for ≥65 years | 700,725 | 524,333 | 838,522 | 1,600,799 | 726,335 |
|  | Due to early death |  | 17,086,727 | 8,293,128 | 6,819,102 | 25,638,979 | 13,534,294 |
| Total cost (excluding productivity loss for ≥65 years) | | | 16,436,325 | 13,549,077 | 17,781,065 | 40,627,001 | 22,893,523 |
| Total cost (including productivity loss for ≥65 years) | | | 34,223,777 | 22,366,537 | 25,438,688 | 67,866,779 | 37,154,152 |

^1)^ USD $1= KRW ₩1,100

**S12 Table. Socioeconomic costs of the five influenza seasons in Koreans with each comorbidity**

|  | | | **Estimated cost (USD)*** | | | | |
| --- | --- | --- | --- | --- | --- | --- | --- |
|  |  |  | **2014/15** | **2015/16** | **2016/17** | **2017/18** | **2018/19** |
| **Diabetes mellitus** | | | | | | | |
| Direct cost | Direct medical cost | | 18,318,288 | 20,021,481 | 21,237,144 | 42,496,127 | 37,297,287 |
|  | Direct medical cost (/person)  Outpatient cost (/person)  Inpatient cost (/person) | | 2,176 | 3,579 | 2,758 | 2,812 | 4,456 |
|  |  |  | 452 | 496 | 451 | 502 | 530 |
|  |  |  | 3,792 | 5,339 | 4,291 | 4,119 | 6,425 |
|  | Direct non-medical cost | Transportation cost | 1,916,192 | 1,721,548 | 1,890,313 | 3,256,640 | 2,169,455 |
|  |  | Nursing cost | 1,759,201 | 1,591,780 | 1,805,613 | 3,627,888 | 2,803,522 |
| Indirect cost | Due to visiting clinic or admission | Excluding productivity loss for ≥65 years | 587,033 | 941,246 | 726,591 | 1,188,570 | 1,409,424 |
|  | Due to early death |  | 2,572,443 | 0 | 13,384,323 | 9,456,556 | 31,410,094 |
|  | Due to visiting clinic or admission | Including productivity loss for ≥65 years | 1,276,881 | 1,424,225 | 1,355,494 | 2,536,788 | 2,251,614 |
|  | Due to early death |  | 26,987,579 | 3,830,454 | 35,742,951 | 50,927,952 | 72,399,344 |
| Total cost (excluding productivity loss for ≥65 years) | | | 25,153,158 | 24,276,056 | 39,043,983 | 60,025,782 | 75,089,782 |
| Total cost (including productivity loss for ≥65 years) | | | 50,258,142 | 28,589,488 | 62,031,514 | 102,845,396 | 116,921,221 |
| **Chronic cardiovascular disease (except hypertension)** | | |  |  |  |  |  |
| Direct cost | Direct medical cost | | 14,156,915 | 10,649,436 | 16,368,949 | 35,620,792 | 28,622,906 |
|  | Direct medical cost (/person)  Outpatient cost (/person)  Inpatient cost (/person) | | 2,471 | 2,958 | 3,170 | 2,973 | 4,966 |
|  |  |  | 441 | 486 | 666 | 525 | 623 |
|  |  |  | 3,757 | 4,286 | 4,635 | 4,476 | 7,202 |
|  | Direct non-medical cost | Transportation cost | 1,580,356 | 1,180,853 | 1,861,800 | 2,839,813 | 1,330,746 |
|  |  | Nursing cost | 1,491,435 | 1,118,067 | 1,867,118 | 3,194,446 | 1,760,752 |
| Indirect cost | Due to visiting clinic or admission | Excluding productivity loss for ≥65 years | 431,926 | 361,554 | 233,912 | 790,239 | 511,434 |
|  | Due to early death |  | 1,758,989 | 545,213 | 0 | 2,090,205 | 9,712,879 |
|  | Due to visiting clinic or admission | Including productivity loss for ≥65 years | 1,032,829 | 790,684 | 1,012,622 | 2,047,184 | 1,128,934 |
|  | Due to early death |  | 22,779,988 | 10,173,665 | 14,842,030 | 30,052,213 | 28,189,809 |
| Total cost (excluding productivity loss for ≥65 years) | | | 19,419,621 | 13,855,123 | 20,331,779 | 44,535,495 | 41,938,718 |
| Total cost (including productivity loss for ≥65 years) | | | 41,041,523 | 23,912,705 | 35,952,519 | 73,754,448 | 61,033,148 |
| **Chronic pulmonary disease** | | |  |  |  |  |  |
| Direct cost | Direct medical cost | | 14,990,052 | 12,512,175 | 16,542,780 | 29,013,808 | 17,889,607 |
|  | Direct medical cost (/person)  Outpatient cost (/person)  Inpatient cost (/person) | | 2,466 | 3,249 | 3,740 | 3,005 | 4,198 |
|  |  |  | 487 | 483 | 613 | 619 | 562 |
|  |  |  | 3,413 | 4,530 | 5,255 | 4,254 | 6,078 |
|  | Direct non-medical cost | Transportation cost | 1,958,172 | 1,351,299 | 2,297,512 | 2,654,109 | 1,132,592 |
|  |  | Nursing cost | 1,861,899 | 1,274,405 | 2,321,945 | 2,965,673 | 1,481,258 |
| Indirect cost | Due to visiting clinic or admission | Excluding productivity loss for ≥65 years | 465,041 | 411,662 | 338,667 | 834,386 | 432,871 |
|  | Due to early death |  | 0 | 3,465,835 | 0 | 2,116,513 | 0 |
|  | Due to visiting clinic or admission | Including productivity loss for ≥65 years | 1,220,727 | 905,409 | 1,290,984 | 1,965,813 | 964,414 |
|  | Due to early death |  | 17,591,572 | 16,341,272 | 8,382,161 | 30,835,274 | 11,627,690 |
| Total cost (excluding productivity loss for ≥65 years) | | | 19,275,164 | 19,015,377 | 21,500,904 | 37,584,490 | 20,936,328 |
| Total cost (including productivity loss for ≥65 years) | | | 37,622,422 | 32,384,561 | 30,835,382 | 67,434,678 | 33,095,561 |
| **Chronic renal disease** | | |  |  |  |  |  |
| Direct cost | Direct medical cost | | 6,367,223 | 7,252,775 | 7,620,181 | 17,342,313 | 11,824,287 |
|  | Direct medical cost (/person)  Outpatient cost (/person)  Inpatient cost (/person) | | 2,164 | 3,470 | 3,580 | 2,955 | 3,959 |
|  |  |  | 401 | 488 | 457 | 446 | 495 |
|  |  |  | 3,571 | 4,725 | 4,892 | 4,353 | 5,988 |
|  | Direct non-medical cost | Transportation cost | 752,571 | 629,642 | 671,388 | 1,239,228 | 709,495 |
|  |  | Nursing cost | 701,048 | 593,513 | 657,260 | 1,380,204 | 898,708 |
| Indirect cost | Due to visiting clinic or admission | Excluding productivity loss for ≥65 years | 341,105 | 291,057 | 286,770 | 519,783 | 535,931 |
|  | Due to early death |  | 813,454 | 2,160,921 | 1,121,500 | 2,564,213 | 0 |
|  | Due to visiting clinic or admission | Including productivity loss for ≥65 years | 579,963 | 494,023 | 510,358 | 1,016,123 | 794,047 |
|  | Due to early death |  | 9,196,172 | 12,130,956 | 10,509,362 | 20,286,888 | 7,076,335 |
| Total cost (excluding productivity loss for ≥65 years) | | | 8,975,401 | 10,927,907 | 10,357,100 | 23,045,741 | 13,968,421 |
| Total cost (including productivity loss for ≥65 years) | | | 17,596,978 | 21,100,909 | 19,968,549 | 41,264,756 | 21,302,871 |
| **Immunocompromised** | | |  |  |  |  |  |
| Direct cost | Direct medical cost | | 10,403,321 | 14,548,652 | 11,414,763 | 29,863,037 | 28,562,814 |
|  | Direct medical cost (/person)  Outpatient cost (/person)  Inpatient cost (/person) | | 2,046 | 3,282 | 2,149 | 2,569 | 4,278 |
|  |  |  | 426 | 395 | 457 | 418 | 449 |
|  |  |  | 3,136 | 4,987 | 3,140 | 3,769 | 6,729 |
|  | Direct non-medical cost | Transportation cost | 1,092,780 | 1,111,623 | 1,102,200 | 2,489,710 | 1,425,293 |
|  |  | Nursing cost | 988,959 | 1,000,443 | 1,037,575 | 2,746,809 | 1,798,070 |
| Indirect cost | Due to visiting clinic or admission | Excluding productivity loss for ≥65 years | 582,458 | 898,902 | 529,546 | 1,470,850 | 1,078,129 |
|  | Due to early death |  | 0 | 8,583,851 | 3,414,705 | 3,303,474 | 28,819,845 |
|  | Due to visiting clinic or admission | Including productivity loss for ≥65 years | 915,689 | 1,133,349 | 867,940 | 2,327,871 | 1,590,129 |
|  | Due to early death |  | 14,862,331 | 19,965,967 | 16,800,861 | 36,692,581 | 63,632,448 |
| Total cost (excluding productivity loss for ≥65 years) | | | 13,067,518 | 26,143,472 | 17,498,789 | 39,873,878 | 61,684,151 |
| Total cost (including productivity loss for ≥65 years) | | | 28,263,080 | 37,760,034 | 31,223,339 | 74,120,007 | 97,008,753 |

^1)^ USD $1= KRW ₩1,100

**S13 Table. Annual average cost per capita (socioeconomic cost) among total and at-risk individuals, assuming no productivity loss for the ≥65 age group**

|  | **Estimated cost (USD)^1)^** | **2014/15** | **2015/16** | **2016/17** | **2017/18** | **2018/19** |
| --- | --- | --- | --- | --- | --- | --- |
| **All adults** | Direct medical cost (/person) | 1,165 (75.9%) | 1,523 (55.3%) | 1,382 (59.7%) | 1,520 (69.6%) | 1,670 (58.6%) |
|  | Direct non-medical cost (/person) |  |  |  |  |  |
|  | Transportation cost | 139 (9.1%) | 143 (5.2%) | 157 (6.8%) | 133 (6.1%) | 106 (3.7%) |
|  | Nursing cost | 113 (7.4%) | 115 (4.2%) | 136 (5.9%) | 133 (6.1%) | 115 (4.0%) |
|  | Indirect cost (/person)^2)^ |  |  |  |  |  |
|  | Due to visiting clinic or admission | 72 (4.7%) | 100 (3.6%) | 69 (3.0%) | 72 (3.3%) | 84 (2.9%) |
|  | Due to early death | 45 (2.9%) | 872 (31.7%) | 572 (24.7%) | 326 (14.9%) | 874 (30.7%) |
|  | Total cost (/person) | 1,534 | 2,753 | 2,316 | 2,184 | 2,848 |
|  | Total cost (national-level)^3)^ | 88,367,860 | 141,314,961 | 107,275,265 | 199,293,934 | 191,544,494 |
| **Sub-population:**  **individuals with comorbidities** | Direct medical cost (/person) | 1,940 (76.7%) | 2,826 (67.9%) | 2,306 (64.0%) | 2,333 (72.1%) | 3,570 (55.8%) |
|  | Direct non-medical cost |  |  |  |  |  |
|  | Transportation cost (/person) | 220 (8.7%) | 264 (6.3%) | 259 (7.2%) | 203 (6.3%) | 208 (3.3%) |
|  | Nursing cost (/person) | 200 (7.9%) | 240 (5.8%) | 247 (6.9%) | 221 (6.8%) | 262 (4.1%) |
|  | Indirect cost (/person)^2)^ |  |  |  |  |  |
|  | Due to visiting clinic or admission | 79 (3.1%) | 146 (3.5%) | 87 (2.4%) | 86 (2.7%) | 128 (2.0%) |
|  | Due to early death | 92 (3.6%) | 684 (16.4%) | 702 (19.5%) | 392 (12.1%) | 2,230 (34.9%) |
|  | Total cost (/person) | 2,530 | 4,160 | 3,601 | 3,236 | 6,398 |
|  | Total cost (national-level)^3)^ | 70,920,160 | 83,091,878 | 83,180,513 | 160,315,665 | 164,075,701 |

^1)^ USD $1= KRW ₩1,100

^2)^ Assuming no loss of productivity for individuals aged 65 and older

^3)^ We extrapolated the social-perspective costs obtained from the eight hospitals nationwide by multiplying the cost by the ratio of the nationwide annual patient visits to that of the eight participating hospitals.

**S14 Table. Data on influenza virus isolates by subtype in South Korea from the Korea Disease Control and Prevention Agency and World Health Organization vaccine composition during the 2014/15–2018/19 seasons**

| Season | Influenza virus isolates, no. (%) | | | | | WHO vaccine composition of trivalent vaccine | Matching status^2)^ | | |
| --- | --- | --- | --- | --- | --- | --- | --- | --- | --- |
|  | Total isolates^1)^ | A (H1N1)  pdm09 | A (H3N2) | A (NS) | B |  | A (H1N1)  pdm09 | A( H3N2) | B |
| 2014/15 | 1,589 (100.0) | 175 (11.0) | 826 (52.0) | 0 (0.0) | 588 (37.0) | A/California/7/2009 (H1N1)pdm09;  A/Texas/50/2012 (H3N2);  B/Massachusetts/2/2012 | Mismatch | Match | Match |
| 2015/16 | 1,312 (100.0) | 582 (44.4) | 61 (4.6) | 1 (0.1) | 668 (50.9) | A/California/7/2009 (H1N1)pdm09;  A/Switzerland/9715293/2013 (H3N2);  B/Phuket/3073/2013 | Match | Match | Mismatch |
| 2016/17 | 1,178 (100.0) | 4 (0.3) | 867 (73.6) | 0 (0.0) | 307 (26.1) | A/California/7/2009 (H1N1)pdm09;  A/Hong Kong/4801/2014 (H3N2);  B/Brisbane/60/2008 | Match | Match | Mismatch |
| 2017/18 | 1,999 (100.0) | 128 (6.4) | 770 (38.5) | 0 (0.0) | 1,101 (55.1) | A/Michigan/45/2015 (H1N1)pdm09;  A/Hong Kong/4801/2014 (H3N2);  B/Brisbane/60/2008 | Match | Match | Mismatch |
| 2018/19 | 1,791 (100.0) | 759 (42.4) | 375 (20.9) | 0 (0.0) | 657 (36.7) | A/Michigan/45/2015 (H1N1)pdm09;  A/Singapore/INFIMH-16-0019/2016 (H3N2);  B/Colorado/06/2017 (B/Victoria/2/87 lineage) | Mismatch | Match | Mismatch |

NS, not subtyped; WHO, World Health Organization; no, number

^1)^The cumulative results from September of the given year to May of the following year are presented.

^2)^The matching rate between the trivalent vaccine and circulating strain in South Korea.

**S1 Figure. Geographic distribution of participating hospitals across South Korea.** The participating hospitals were distributed across key regions of South Korea, including the capital metropolitan area (Seoul, Incheon, and Gyeonggi), Gangwon Province, Jeonnam Province, and Daegu, thereby ensuring nationwide geographic coverage. The greater representation of hospitals in the capital region is consistent with the population distribution, as over 50% of the Korean population resides in this area.

**
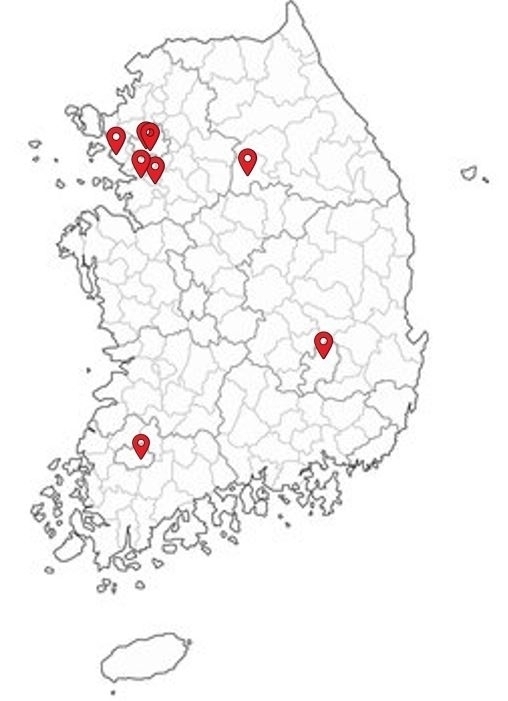
**

**S2 Figure. Estimated incidence per 100,000 persons of laboratory-confirmed influenza and related morbidity and mortality during study: total population (overall, 20–64, ≥65 years)^1)^, subpopulation of at-risk individuals (overall, 20–64, ≥65 years)^2)^, and individuals with each comorbidity^3)^**


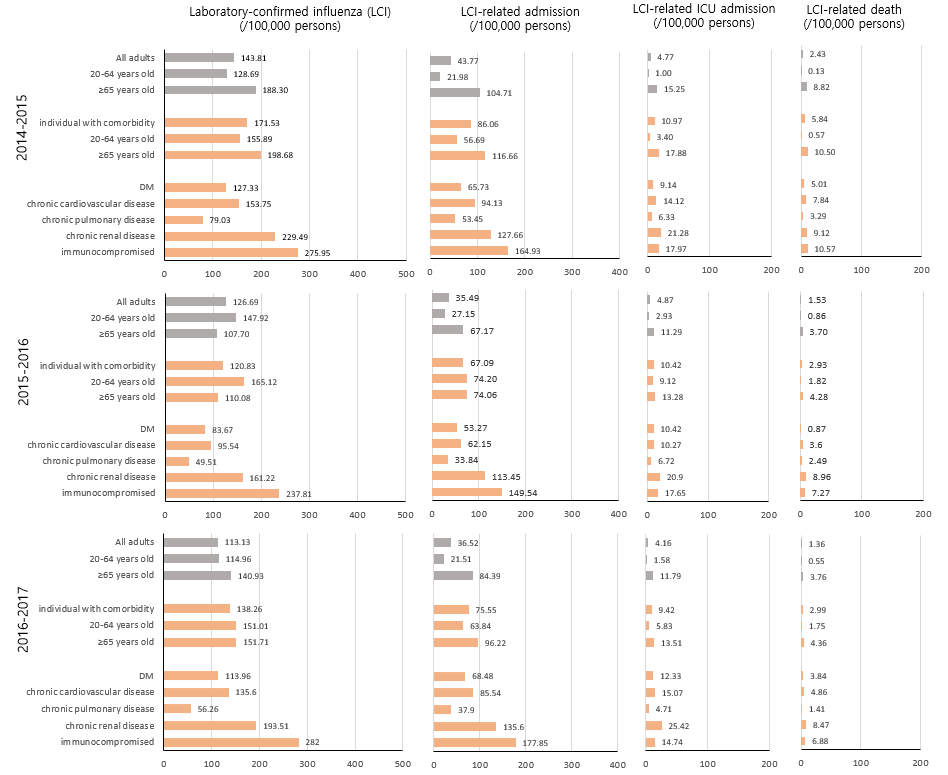


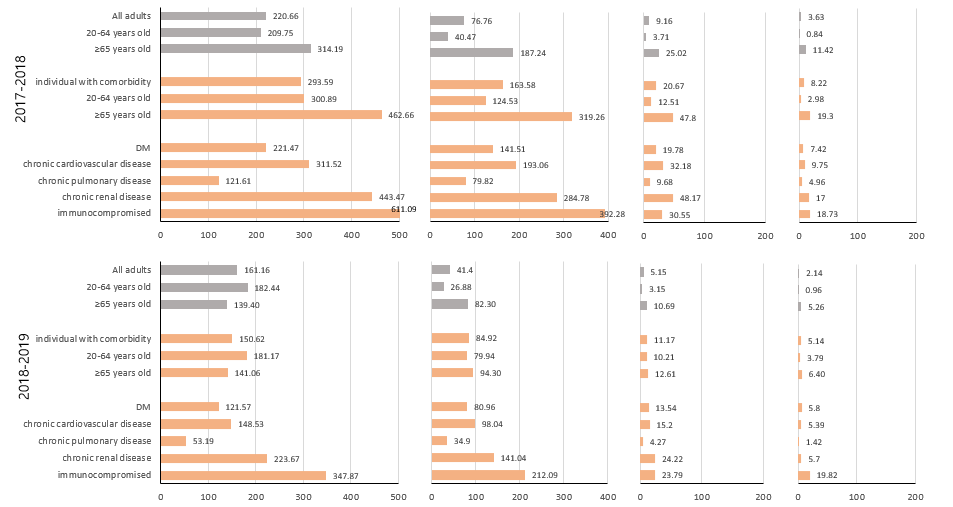


ICU, intensive care unit; DM, diabetes mellitus

^1)^ For the entire adult population and for each age group of adults, calculations were based on the demographic data provided by Statistics Korea as the denominator.(4)

^2)^ The denominator for the overall adult population and age-specific groups considered the prevalence of underlying health conditions. Based on a review of reports and literature in South Korea, the prevalence of individuals with one or more comorbidities among the overall adult population, those aged 20–64, and those aged 65 years and older was determined to be 40.8%, 23.5%, and 82.2%, respectively,.(5, 6) Owing to limitations in age-specific data, age groups were classified into two categories (20–64, 65 years, and older).

^3)^ The denominator for the overall adult population and age-specific groups considered the prevalence of each underlying health condition. The prevalence rates applied were 16.5% for diabetes, 9.3% for chronic cardiovascular diseases (excluding hypertension), 19.2% for chronic pulmonary diseases, 3.2% for chronic kidney diseases, and 4.6% for immunodeficiency.(6) However, caution is needed when interpretating these values, as the cited paper estimated the number of individuals with comorbidity based on ICD-10 codes from claims data. For instance, in the context of healthcare practices in South Korea, there is a tendency to overuse asthma codes (e.g., using diagnostic codes for nebulizer prescription claims), which could potentially result in an overestimation of the proportion of chronic pulmonary diseases.

**S3 Figure. Annual average cost per capita (direct medical cost only), stratified by age and comorbidity**


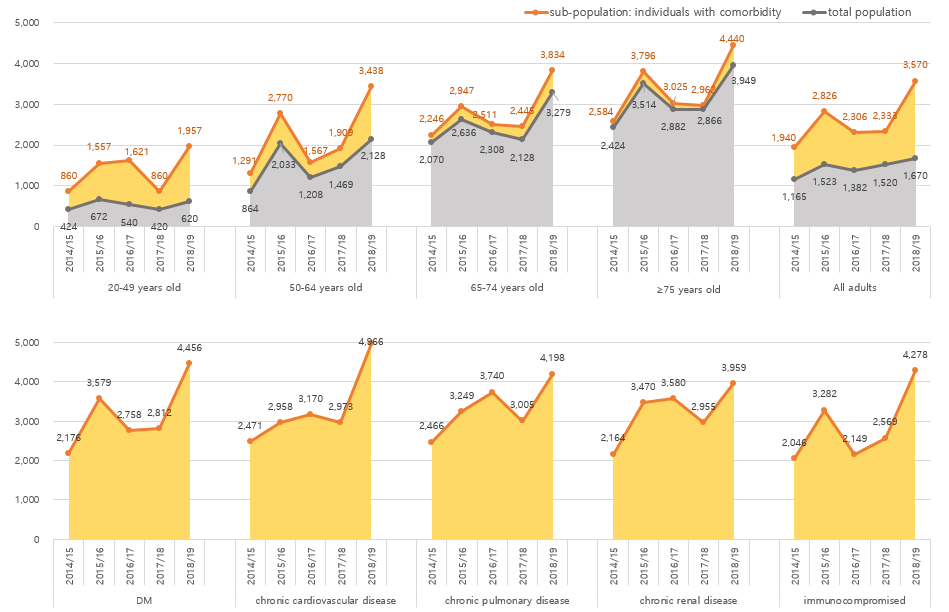


**S4 Figure. Annual average cost per capita (socioeconomic cost): analysis based on each comorbidity, assuming that productivity loss occurs across all age groups**

**S5 Figure. Annual average cost per capita (socioeconomic cost), stratified by age and comorbidity, assuming no productivity loss for individuals aged 65 and older**


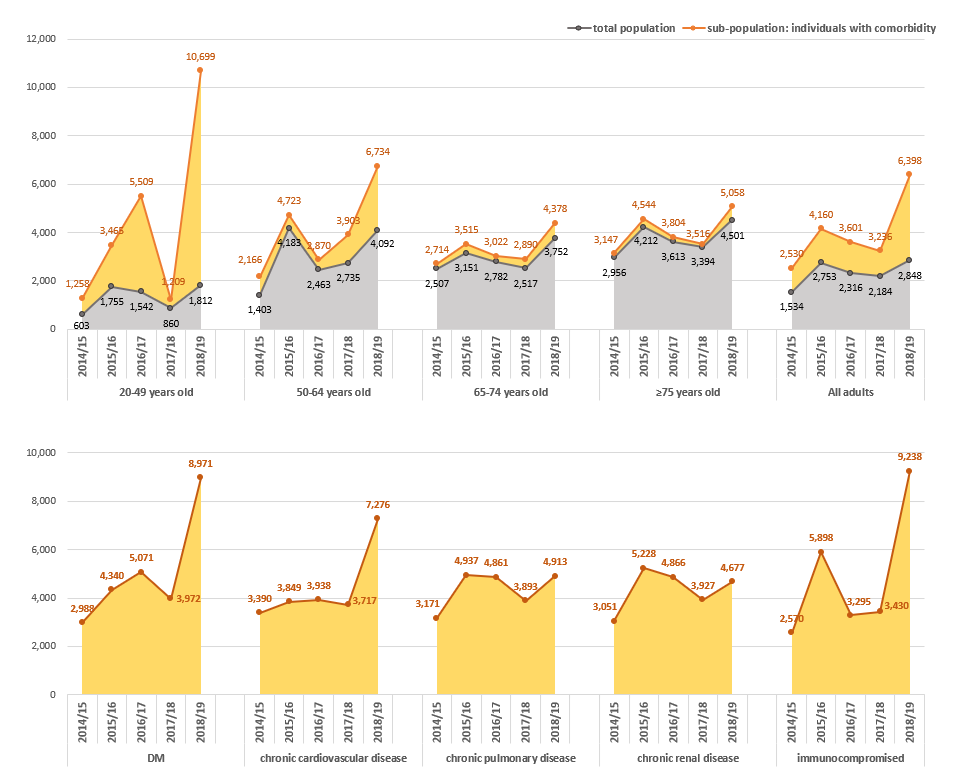


**References**

1. Choi WS, Cowling BJ, Noh JY, et al. Disease burden of 2013-2014 seasonal influenza in adults in Korea. PLoS One, **2017**; 12: e0172012.

2. Korea Health Panel Survey. The Report on the Korea Health Panel Survey of 2018. Available at: <https://www.khp.re.kr:444/web/research/board/list.do?bbsid=13> (Accessed February 4, 2024).

3. Empolyment and Labor Statistics. Available at: <http://laborstat.moel.go.kr/hmp/tblInfo/TblInfoList.do?menuId=0010001100101&leftMenuId=0010001100101&bbsId> (Accessed Jan 21, 2024).

4. Korea Statistical Information Service. Available at: <https://kosis.kr/statisticsList/statisticsListIndex.do?vwcd=MT_ZTITLE&menuId=M_01_01> (Accessed: Jan 25, 2024).

5. Seo JH. Analysis of multiple chronic disease types and healthcare costs using data from the Korea Health Panel. Health and Welfare Forum 2021; 2021(12): 17-28.

6. Yoon JG, Kim Y-E, Choi MJ, et al. Herpes Zoster reactivation after mRNA and adenovirus-vectored Coronavirus Disease 2019 vaccination: analysis of National Health Insurance database. J Infect Dis, **2023**; 228: 1326-1335.
